# Design, Synthesis and Anticancer Evaluation of Nitroimidazole Radiosensitisers

**DOI:** 10.3390/molecules28114457

**Published:** 2023-05-31

**Authors:** Lydia P. Liew, Avik Shome, Way W. Wong, Cho R. Hong, Kevin O. Hicks, Stephen M. F. Jamieson, Michael P. Hay

**Affiliations:** 1 Auckland Cancer Society Research Centre, The University of Auckland, Auckland 1023, New Zealand; l.liew@auckland.ac.nz (L.P.L.); a.shome@auckland.ac.nz (A.S.); w.wong@auckland.ac.nz (W.W.W.); c.hong@auckland.ac.nz (C.R.H.); k.hicks@auckland.ac.nz (K.O.H.); 2 Maurice Wilkins Centre for Molecular Biodiscovery, The University of Auckland, Auckland 1010, New Zealand; 3 Department of Ophthalmology, The University of Auckland, Auckland 1023, New Zealand; 4 Department of Pharmacology and Clinical Pharmacology, The University of Auckland, Auckland 1023, New Zealand

**Keywords:** chemoradiotherapy, DNA damage, electron affinity, hypoxia, nitroimidazole, prodrugs, radiosensitisers, radiotherapy, sulfonamide, tumour microenvironment

## Abstract

The role of hypoxic tumour cells in resistance to radiotherapy, and in suppression of immune response, continues to endorse tumour hypoxia as a bona fide, yet largely untapped, drug target. Radiotherapy innovations such as stereotactic body radiotherapy herald new opportunities for classical oxygen-mimetic radiosensitisers. Only nimorazole is used clinically as a radiosensitiser, and there is a dearth of new radiosensitisers in development. In this report, we augment previous work to present new nitroimidazole alkylsulfonamides and we document their cytotoxicity and ability to radiosensitise anoxic tumour cells in vitro. We compare radiosensitisation with etanidazole and earlier nitroimidazole sulfonamide analogues and we identify 2-nitroimidazole and 5-nitroimidazole analogues with marked tumour radiosensitisation in ex vivo assays of surviving clonogens and with in vivo tumour growth inhibition.

## 1. Introduction

Radiotherapy (RT) plays a central role in cancer treatment with ~50% of all cancer patients receiving RT, either in a definitive setting (high-dose fractionated RT with curative intent), as adjuvant therapy after surgery, or as palliation to relieve symptoms of disease [1,2]. The combination of RT with chemotherapy (CRT) has emerged as the standard of care for definitive RT in many tumour types (e.g., cisplatin in head and neck squamous cell carcinoma (HNSCC) [3]), but chemoradiotherapy is limited by normal tissue toxicities both within and beyond the radiation field [4]. Key to further advances in RT will be exploitation of the tumour microenvironment to identify drug targets that will provide tumour-selective radiosensitisation [2,5,6,7].

A key aspect of the tumour microenvironment that limits the therapeutic effectiveness of RT is hypoxia [8,9], a common characteristic of tumours, owing to the disordered and dysfunctional tumour vasculature that supports tumour growth [8,10]. Higher radiation doses (from two- to three-fold) are required to kill hypoxic cells compared to well-oxygenated cells, since radiation requires oxygen to induce DNA damage [11]. Tumour hypoxia is prevalent in HNSCC tumours [12] and there is compelling evidence that patients with hypoxic human papilloma virus (HPV)-ve HNSCC tumours have inferior responses following RT or CRT [13,14,15,16].

Efforts to target tumour hypoxia have included the use of oxygen-mimetic radiosensitisers, where low molecular weight compounds with an electron affinic nitro group can mimic oxygen by reacting with DNA radicals produced by RT [11]. Several nitroimidazole radiosensitisers have been evaluated clinically, including misonidazole (**1**) [17,18,19,20], etanidazole (**2**) [21,22,23] and nimorazole (**3**) [24,25]. Misonidazole (**1**) and etanidazole (**2**) were both active, but treatment was associated with significant toxicities, particularly, in combination with fractionated RT [19,20]. Nimorazole (**3**) is better tolerated [26], but it is a relatively weak sensitiser [24] because of its low electron affinity [27,28,29]. Nimorazole (**3**) is active in combination with fractionated RT for HNSCC [25] and is in clinical use in Denmark. The more polar doranidazole (**4**) has undergone investigation in non-small cell lung cancer [30] and pancreatic cancer in conjunction with intra-operative RT [31] and displayed a small survival advantage [32].

**Figure 1:**
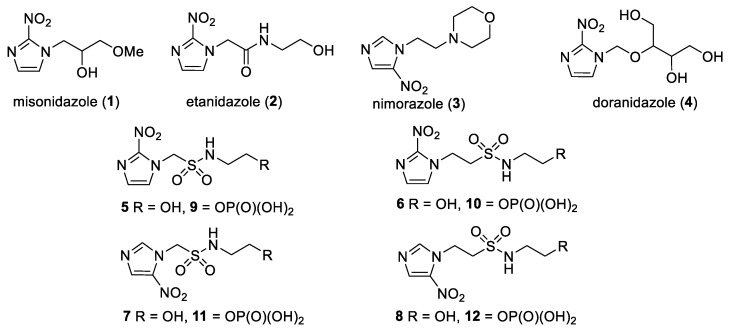
Nitroimidazole radiosensitisers.

We have previously reported our efforts to develop a novel class of nitroimidazole sulfonamide radiosensitiser that incorporated both highly electron-affinic compounds that act as potent sensitisers through reacting with DNA radicals and bioreduction to cytotoxic species, as well as less electron-affinic compounds that are less effective sensitisers but better tolerated [33,34]. We identified four compounds (**5**–**8**) that effectively sensitised HCT116 colon carcinoma cells and tumours to radiation but, due to their relatively low solubility, required formulation as phosphate prodrugs (**9**–**12**) [34] (Figure 1). Here, we extend our investigation of these nitroimidazole sulfonamide radiosensitisers to characterise a wider compound set in additional cell lines, including two HNSCC cell lines, and to identify effective compounds that do not require a prodrug approach in vivo.

## 2. Results

### 2.1. Synthesis

**Scheme 1:**
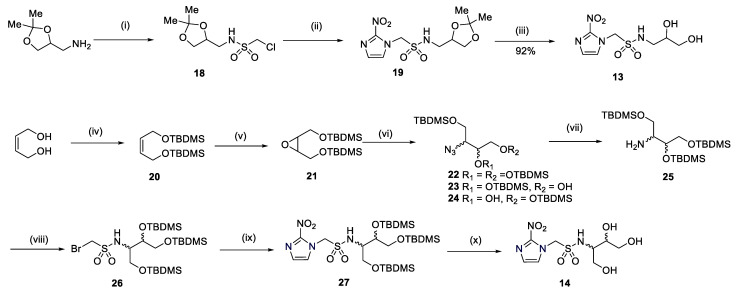
Synthesis of new nitroimidazole methyl sulfonamides. Reagents: (i) ClCH_2_SO_2_Cl, Et_3_N, DCM, 81%; (ii) 2-nitroimidazole, NaI, Cs_2_CO_3_, DMF, 42%; (iii) HOAc/H_2_O/THF, 92%; (iv) TBDMSCl, imidazole, DMF, 97%; (v) MCPBA, DCM, 96%; (vi) NaN_3_, NH_4_Cl, DMF, 25%; (vii) H_2_, Pd/C, 100%; (viii) BrCH_2_SO_2_Cl, Et_3_N, DCM, 54%; (ix) 2-nitroimidazole, NaI, Cs_2_CO_3_, DMF, 54%; (x) TBAF, THF, 61%.

In our earlier investigation, we evaluated 33 novel nitroimidazole sulfonamide radiosensitiser compounds for in vitro cytotoxicity and radiosensitisation in HCT116 cells [33,34]. We identified four candidate alcohols **5**–**8** with promising in vitro activity, but low aqueous solubility required formulation as the corresponding phosphate prodrugs **9**–**12** for in vivo evaluation (Figure 1). A further five compounds (**13**–**17**) were synthesized to explore the use of diol (**13**, **15**), triol (**14**) and morpholine groups (**16**) to increase aqueous solubility, thus, avoiding the use of phosphate prodrugs (Scheme 1). We also explored the effect of reversing the orientation of the sulfonamide linker (**17**). The use of diols and triols as neutral solubilising groups have ample precedent in the development of doranidazole [35,36].

Reaction of chloromethanesulfonyl chloride with the commercially available acetonide gave a good yield of chloride **18**, and alkylation of 2-nitroimidazole yielded sulfonamide **19** which was deprotected to give the diol **13** (Scheme 1). Silylation of the commercially available *cis*-butene-1,4-diol followed by epoxidation readily afforded the oxirane **21** in excellent yield [37]. Ring opening with azide serendipitously gave the trisilyl ether **22**, as well as the alcohol **23** from a complex mixture, rather than the expected azido alcohol **24**. Reduction of the azide **22** gave amine **25** which was condensed with bromomethyl sulfonyl chloride to give the bromide **26**. Alkylation of 2-nitroimidazole gave sulfonamide **27** which was readily deprotected to give the triol **14**.

**Scheme 2:**
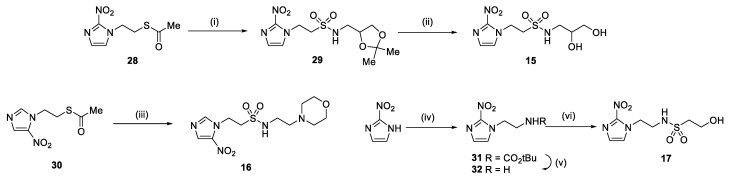
Synthesis of new nitroimidazole ethyl sulfonamides. Reagents: (i) NCS, HCl, MeCN, then (2,2-dimethyl-1,3-dioxolan-4-yl)methanamine, iPr_2_NEt, DCM, 24%; (ii) HOAc/H_2_O/THF, 85%; (iii) NCS, HCl, MeCN, then 4-(2-aminoethyl)morpholine, iPr_2_NEt, DCM, 24%; (iv) *tert*-butyl (2-bromoethyl)carbamate, NaI, Cs_2_CO_3_, DMF, 67%; (v) TFA, 98%; (vi) mercaptoethanol, NCS, nBu_4_NCl, water, then **32**, iPr_2_NEt, MeCN, 22%.

The thioester **28** [34] was converted to the corresponding sulfonyl chloride and reacted with the amino acetonide to give sulfonamide **29** which was deprotected to give diol **15** (Scheme 2). Similarly, conversion of thioester **30** to sulfonyl chloride in situ and reaction with 4-(2-aminoethyl)morpholine gave sulfonamide **16**. The reversed sulfonamide was prepared by alkylation of 2-nitroimidazole with a BOC-protected aminoethyl bromide. Deprotection and reaction with the sulfonyl chloride derived from mercaptoethanol in situ [38] gave sulfonamide **17** directly.

### 2.2. Physicochemical Properties

The use of a diol sidechain for 2-nitroimidazole **13** provided a large increase (>five-fold) in aqueous solubility compared to the corresponding alcohol **5** (18 mM) [34]; however, addition of another alcohol group in triol **14** did not increase solubility (Table 1). For the isomeric 5-nitroimidazole **15,** little additional solubility was gained compared to **6** (75 mM). The use of a charged morpholine group in 5-nitroimidazole **15** provided a modest increase in solubility compared to analogous alcohol **7** (23 mM). Curiously, the reversed sulfonamide **17** provided an increase in solubility compared to the conventional sulfonamide analogue **6**. Overall, the use of polyols did not provide increased aqueous solubility compared to the phosphate prodrug approach but did provide sufficient aqueous solubility to allow full evaluation of the analogues. The compounds were all stable under cell culture conditions. The electron affinity of the compounds was assessed by comparison to measured one-electron reduction potentials measured for analogues of **5**–**8** [34] and literature values [28]. Typically, 2-nitroimidazoles provide reduction potentials in the range from −380 to −400 mV and the proximity of the electron withdrawing sulfonamide substituent can increase this to ca. from −340 to −350 mV for a C-1 linkage, with a minimal increase (ca. 10 mV) for a C-2 linkage [34]. Similarly, the one electron reduction potentials for 5-nitroimidazoles (e.g., **3**) typically lie in the range from −440 to −460 mV, with 2-methyl-5-nitroimidazole analogues as low as −500 mV, and the addition of a C-1 or C-2 sulfonamide substituent increases this by ca. 25 and 10 mV, respectively [34].

### 2.3. In Vitro Cytotoxicity

The new compounds **13**–**17** were compared to the clinical compounds misonidazole (**1**), etanidazole (**2**) and nimorazole (**3**), the recently described alcohols **5**–**8**, as well as a representative set of nitroimidazole sulfonamides **33**–**46** [34]. Compounds were tested for cytotoxicity (as IC_50_) after 4 h of drug exposure under both oxic and anoxic conditions with a five-day regrowth period. This allowed derivation of hypoxia cytotoxicity ratios (HCR) as IC_50(oxic)_/IC_50(anoxic)_ in an HCT116 subclone HCT116/54C and two HNSCC cell lines, FaDu and UT-SCC-74B (Table 2). The clinical radiosensitisers **1**–**3** and the lead nitroimidazole sulfonamides **5**–**8** demonstrated increased cytotoxicity under anoxia relative to oxia consistently across the cell line panel, with more electron affinic 2-nitroimidazoles showing greater HCR values than the corresponding 5-nitroimidazole analogues, for example, comparing **1**, **2** and **5** with **3** and **7**. Similarly, 2-nitroimidazole **6** was more potent under anoxia leading to increased HCR values across cell lines. The new polyol 2-nitroimidazole analogues **13** and **14** were ca. 10-fold less potent under anoxia than **5**, while **15** was ca. 5-fold less potent than **6** under anoxia, leading to reduced HCR values for these 2-nitroimidazoles. The 5-nitroimidazole **15** displayed low oxic potency and little hypoxic selectivity consistent with its lower electron affinity. Reversal of the sulfonamide group in compound **17** made little impact on oxic potency or hypoxic selectivity compared to the original orientation in sulfonamide **6**. Preparation of **16** allowed a comparison of the effect of a weakly basic amine sidechain across the four nitroimidazole analogues (**34**, **36**, **38** and **16**) with their corresponding alcohols **5**–**8** in vitro and their phosphate prodrugs **9**–**12** in vivo. The increased solubility of the morpholines compared to the corresponding alcohols allowed determination of HCR values across the panel. The HCR values of the morpholines were broadly similar to the corresponding alcohols (e.g., **5** compared to **34**, etc.) and tracked with nitroimidazole electron affinity.

Evaluation of the broader set of nitroimidazole sulfonamides **33**–**46** reinforced the previous observations, with anoxic potency being primarily a function of electron affinity with contributions from amine pKa and lipophilicity [34]. Thus, 2-nitroimidazoles **33** and **34** were strongly hypoxia selective with attenuated selectivity observed for the corresponding analogues **35** and **36** because of reduced electron affinity. This change reflects the reduced influence of the electron withdrawing sulfonamide group with a 2-carbon linker. Similarly, in the 5-nitroimidazole series, the C-2 linked analogue **39** displayed lower hypoxic selectivity than the C-1 linked analogues **37** and **38**. Likewise, addition of a weakly electron-withdrawing 2-methyl substituent produced low anoxic potency and hypoxic selectivity across the series **40**–**44,** with only the addition of more basic amine groups in **45** and **46** modestly increasing anoxic potency and hypoxic selectivity. The set of compounds recapitulated the observations from previous work that the more electron affinic 2-nitroimidazoles are likely to display both hypoxic selectivity and oxygen-mimetic radiosensitisation, although this may be mitigated by low pKa and lipophilicity as seen for **13**–**15**. As expected, the cytotoxic potency and hypoxic selectivity of the analogues were consistent between cell lines.

### 2.4. In Vitro Radiosensitisation

Next, we evaluated in vitro radiosensitisation by using a representative subset of nitroimidazole compounds in HCT116/54C cells with a clonogenic survival endpoint. Radiosensitisation was determined by treating cells under anoxia with a range of radiation doses alone and with compounds at equimolar doses (1 mM). Sensitiser enhancement ratios (SER) were calculated as the ratio of radiation dose required for 1% clonogenic survival alone or with compound (Table 3). The SER values were strongly influenced by the electron affinity of the nitroimidazole, and this was modulated by the influence of the side chain. The more electron affinic 2-nitroimidazoles were more effective sensitisers, for example, **5** compared to **7** and **6** compared to **8**. Increased chain length reduced the electron withdrawal of the sulfonamide group and consequently reduced SER values, for example, **5** compared with **6** and **7** compared with **8**. The character of the sidechain also influenced radiosensitisation with reduced lipophilicity resulting in reductions in SER values. This was most dramatically seen in the loss of activity observed for diol **13** and triol **14** compared to alcohol **5**. Addition of the weakly basic morpholine sidechain provided increased activity, for example, **16** compared with **8**, **34** compared with **5**, **36** compared with **6**; two 5-nitroimidazole examples provided similar sensitisation, for example, **38** and **42** compared to **7** and **41**, respectively. Overall, it was clear that the new analogues did not provide enhanced sensitisation compared to existing analogues and that compounds with a morpholine solubilising side chain provided sufficient solubility [34] to explore the most active 2-nitroimidazole **34** and 5-nitroimidazole **38** sensitisers further in vivo.

### 2.5. In Vivo Pharmacokinetics

The most promising 2-nitroimidazole **34** and 5-nitroimidazole **38** compounds were selected for in vivo evaluation on the basis of solubility, in vitro cytotoxicity and radiosensitisation data. These compounds were both well tolerated by i.v. injection in NIH-III mice at their maximum achievable dose (MAD) of 2.2 mmol/kg. This MAD was also achieved for some of our earlier compounds, but only when we employed a phosphate prodrug strategy owing to their poor solubility [34]. Pharmacokinetic evaluation of 2-nitroimidazole **34** achieved a maximum concentration (C_max_) of 1.53 mM 5 min after dosing with a terminal half-life of 1.52 h and AUC of 0.96 mM·h (Table 4). The C_max_ and AUC exceeded that achieved for earlier phosphate prodrugs **9**–**12**, but was less than **2**. On this basis we advanced both morpholine analogues into in vivo radiosensitisation studies.

### 2.6. In Vivo Radiosensitisation

**Figure 2:**
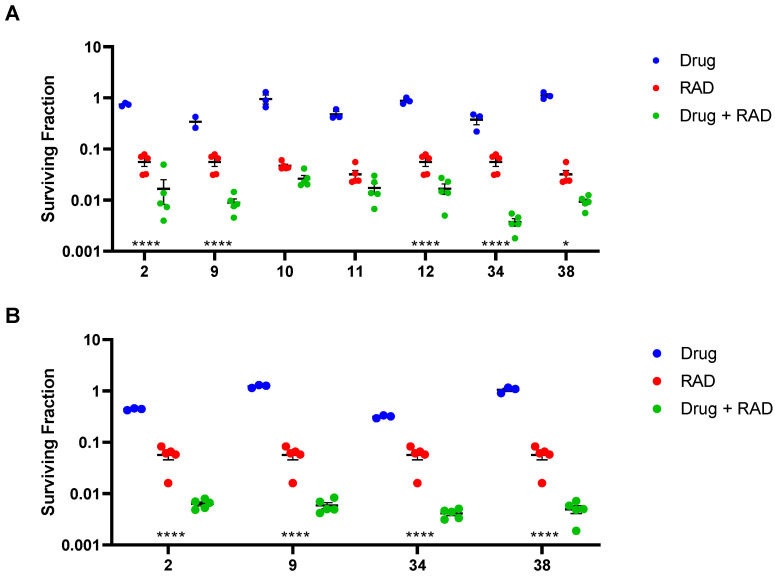
In vivo radiosensitisation of hypoxic cells in (**A**) HCT116/54C tumours and (**B**) UT-SCC-74B tumours by ex vivo clonogenic assay in NIH-III mice. Tumours were excised 18 h after mice were treated by i.v. injection with 2.2 mmol/kg drug alone, 10 Gy radiation (RAD) alone or 2.2 mmol/kg drug combined with 10 Gy radiation (*n* = 3–5), and plated to determine the number of surviving cells relative to untreated controls. ****, *p* < 0.0001; *, *p* < 0.05 for comparison of RAD vs. drug + RAD by one-way ANOVA with Sidak’s multiple comparison analysis.

Compounds **34** and **38** were evaluated in an ex vivo clonogenic assay using HCT116/54C tumour xenograft model as compared with **2** and our four phosphate prodrugs **9**–**12** that had previously demonstrated activity in the same assay in HCT116 parental tumours [34]. Compounds **34** and **38** both significantly enhanced the tumour killing of radiotherapy by targeting the hypoxic cells that radiation spares (Figure 2A), as did **2**, **9** and **12**, but not **10** and **11**. The 2-nitroimidazole **34** had an in vivo sensitisation ratio (SR = surviving fraction after radiation/surviving fraction after drug + radiation) of 14.9 in HCT116/54C tumours, which was superior to that of **2** and the phosphate prodrugs **9**–**12** (Table 5). The SR of **38** was 3.43, which was comparable to **2**, but 1.8-fold lower than **9**. A second tumour model, UT-SCC-74B, was used to confirm in vivo radiosensitisation. All four compounds tested (**2**, **9**, **34** and **38**) significantly enhanced tumour cell killing by radiotherapy, with slightly higher SR values of 13.8 and 11.5 for **34** and **38**, respectively, than those seen with **2** and phosphate prodrug **9** (Figure 2B, Table 5). Evidence of single agent activity was observed for **34** in the ex vivo clonogenic assays with 62.3 ± 7.9% cell kill observed in HCT116/54C tumours and 68.4 ± 1.2% cell kill in UT-SCC-74B tumours (Figure 2) relative to untreated tumours (*p* < 0.005 and *p* < 0.0001, respectively). Among the other compounds evaluated, only **9** (65.7 ± 8.2% cell kill; *p* < 0.005) in HCT116/54C tumours and **2** (55.9 ± 1.1% cell kill; *p* < 0.0005) in UT-SCC-74B tumours demonstrated significant single agent activity.

**Figure 3:**
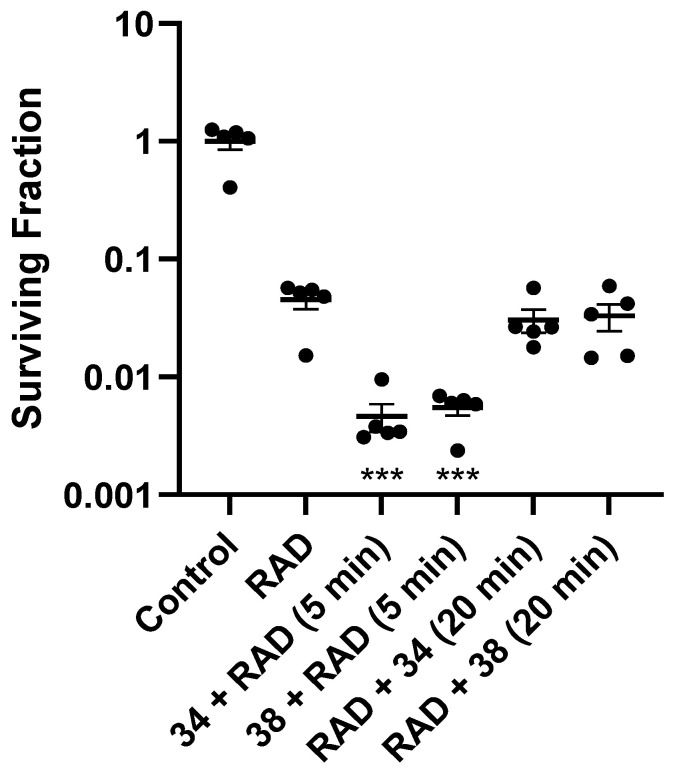
Ex vivo clonogenic assay in HCT116/54C tumours from NIH-III mice treated with **34** or **38** at 2.2 mmol/kg by i.v. injection either 5 min before 10 Gy radiation (RAD) or 20 min after (*n* = 5). Tumours were collected 18 h after treatment and plated to determine the number of surviving cells relative to untreated controls. ***, *p* < 0.001 vs. RAD alone by one-way ANOVA with Sidak’s multiple comparison analysis.

Since nitroimidazoles may possess dual mechanisms of cell killing (both electron-affinic oxygen mimetic radiosensitisation and hypoxia-selective cytotoxicity), we evaluated the mechanism of action for **34** and **38**. Oxygen-mimetic sensitisers must be present at the time of irradiation to work, whereas hypoxia-selective cytotoxins may exert their effect even when given after radiation. Drugs were dosed either 5 min before radiation or 20 min after radiation in mice with HCT116/54C tumours in the ex vivo clonogenic assay. Compounds **34** and **38** both significantly enhanced radiation cell kill when administered 5 min before radiation with SR values of 9.76 and 8.26, while no significant additional killing over radiation was observed when given after radiation (SR values of 1.49 and 1.38, respectively, Figure 3). This confirms their major mode of action as oxygen-mimetic radiosensitisers rather than hypoxia-selective cytotoxins.

### 2.7. Inhibition of Tumour Growth

**Figure 4:**
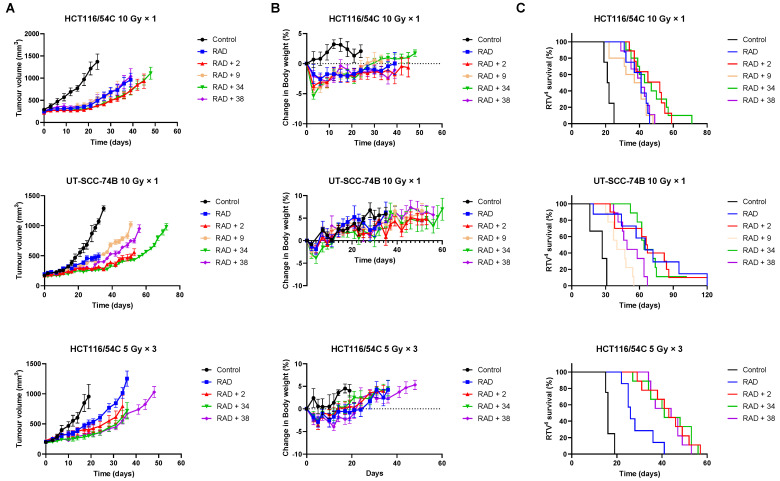
Tumour growth inhibition in HCT116/54C and UT-SCC-74B tumour xenografts. NIH-III mice were treated with radiosensitisers at 2.2 mmol/kg by i.v. injection, 5 min prior to radiation, given either as a single dose at 10 Gy or fractionated over 3 successive weeks (day 0, 7 and 14) at 5 Gy. (**A**) Average tumour volume over time (±SEM); (**B**) change in bodyweight (mean ± SEM); (**C**) RTV^4^ survival time. *N* = 8–10 for treatments and 3–4 for controls.

The ability of **34** and **38** to inhibit tumour growth was evaluated in HCT116/54C and UT-SCC-74B xenograft models and compared to **2** and phosphate prodrug **9**. In mice with HCT116/54C and UT-SCC-74B tumours, a single dose of **34** was able to sensitise 10 Gy radiation to a similar level observed with **2**, while **38** and **9** had similar activity to radiation alone (Figure 4A). In a further comparison using a fractionated radiation schedule (qw × 3, 5 Gy) in HCT116/54C tumours, both **34** and **38** delayed tumour growth relative to radiation alone, with similar activity to **2**. All treatments were well tolerated in both tumour models with no or minor reductions in bodyweight compared to radiation alone (Figure 4B). On evaluation of survival analysis, assessed as time for tumours to quadruple in size following treatment initiation (RTV^4^, relative tumour volume 4×), **2**, **34** and **38** prior to radiation significantly increased survival compared to radiation alone (*p* < 0.05), but only in HCT116/54C tumours treated with fractionated radiation (Figure 4C). There was no significant difference in activity between any of the radiosensitisers in any tumour model.

## 3. Discussion

The development of effective radiosensitisers has been of increasing interest in recent years [5,6,7]. One of the key recommendations from these consensus statements has been that the addition of sensitisers to radiation treatment schedules should not exacerbate normal tissue toxicities because the therapeutic ratio has been titrated to a maximum with little tolerance in normal tissues for further toxicity. Oxygen-mimetic nitroimidazole radiosensitisers are not a new idea, but evolving treatment paradigms present a new opportunity for this class. Nitroimidazole radiosensitisers are designed to oxidise DNA radicals produced by radiation and to cause DNA strand breaks analogously to oxygen [11]. This physicochemical process is largely independent of cell type or genetic background, and it signals the opportunity to expand the use of these radiosensitisers into other tumour types and treatment modalities where RT is limited by hypoxia. Importantly, the rate of reaction with DNA radicals is considerably less than oxygen, limiting their activity to hypoxic tissue and increasing the effective radiation dose to a treatment-resistant compartment without creating additional toxicities.

Having identified a new chemical class of nitroimidazole radiosensitisers where a sulfonamide linking group provides novelty and modulates the nitroimidazole electron affinity, we have taken the opportunity to design new radiosensitisers that build on known structure activity relationships and discover lead compounds with clinical potential. Although we had previously identified effective radiosensitisers in **5**–**8**, these required a phosphate prodrug approach to provide compounds (**9**–**12**) sufficiently soluble for in vivo use [34]. Here, we explored the use of polyol sidechains **13**–**15** to enhance aqueous solubility as demonstrated for **4** and related compounds [39]. We also explored reversal of the key sulfonamide group in **17** and, with compound **16**, completed an analogous set of morpholine analogues (**34**, **36**, **38** and **16**) to the effective alcohols **5**–**8**, respectively. The use of polyol or morpholine sidechains provided useful increases in aqueous solubility, but aqueous solubilities were considerably less than the phosphates **9**–**12** [34]. The polyols **13**–**15** showed weak hypoxic selectivity (Table 2) and were ineffective as radiosensitisers (Table 3). Reversing the sulfonamide moiety in **17** made little difference in either hypoxic selectivity or radiosensitisation compared to the analogous **6**. Comparison of the weakly basic amines **34**, **36**, **38** and **16** with their corresponding alcohols **5**–**8** in vitro showed increased solubility of the morpholines compared to the corresponding alcohols, which was sufficient to allow determination of HCR values across the panel. The HCR values of the morpholines were broadly similar to the corresponding alcohols (e.g., **5** and **34**, etc.) and tracked with nitroimidazole electron affinity. When evaluated as radiosensitisers in cells at equimolar concentrations, the SER values tracked with electron affinity with **34** and **36** showing strong radiosensitisation. The morpholine analogues showed increased (**34**, **36** and **16**) or similar (**38**) radiosensitisation compared to the alcohols **5**, **6**, **8** and **7**, respectively.

We elected to advance **34** and **38** to in vivo evaluation as the best examples of 2- and 5-nitroimidazole analogues, respectively. The compounds were both well tolerated by NIH-III mice and could be given at a MAD of 2.2 mmol/kg, allowing comparison with **2** and the phosphate prodrugs **9**–**12** at equimolar doses. We evaluated **2**, **9**–**12** and **34** and **38** using an ex vivo excision assay to determine how well they sensitised HCT116/54C tumour xenografts to a single 10 Gy dose of radiation. All compounds provided significant additional cell killing in combination with radiation with the 2-nitroimidazoles **2**, **9** and **34** giving strong SR values. Most compounds were inactive as a single agent, except for a small amount of cell killing for 2-nitroimidazoles with high electron affinity in the HCT116/54C (**9** and **34**) and UT-SCC-74B (**2** and **34**) models, suggesting that hypoxia-selective cytotoxicity might have contributed to the sensitising ability of these compounds. To further explore the potential contribution of hypoxia-selective killing to radiosensitisation, we compared **34** and **38** when given 5 min before radiation and 20 min after radiation. When given after radiation, the compounds cannot participate in the oxygen mimetic sensitisation process and so any additional cell killing reflects the contribution from hypoxia-selective cytotoxicity to tumour cell killing. Compounds **34** and **38** provided ca. one log of additional cell killing when given 5 min prior to radiation, and they provided a small (but nonsignificant) amount of additional cell killing compared to radiation when given post radiation. While **34** did show substantial HCR values in vitro (HCR values of 23–38) and some single agent activity in ex vivo excision assays, the small effect seen post-irradiation in this experiment suggests that oxygen-mimetic radiosensitisation is the dominant mechanism for the two nitroimidazoles **34** and **38**.

One of the limitations in the clinical development of previous radiosensitisers was use with fractionated RT, where fractionation of the radiation dose is designed to allow tumour reoxygenation between radiation doses, thus, reducing the potential for radiosensitisation [40,41,42]. Administering a dose of radiosensitiser with each fraction of radiation was unachievable for misonidazole and etanidazole due to cumulative peripheral neurotoxicity [43,44]. The growing use of stereotactic body radiotherapy (SBRT), which delivers hypo-fractionated (1–8 doses), high-dose (25–60 Gy total dose) radiation to treat tumours [45,46], offers new opportunity for radiosensitisers in hypo-fractionated schedules. This new approach leverages recent advances in the accuracy and precision of radiation delivery to allow dose intensification to small tumours while minimising the effects to adjacent normal tissue. Clinical trials using SBRT to treat various solid tumours have demonstrated comparable control, toxicity and efficacy profiles to fractionated RT [46,47,48], and SBRT is being extensively explored in primary and recurrent HNSCC [49,50,51]. The reduced treatment time and number of patient visits, combined with emerging potential to replace surgery with an outpatient procedure, indicates substantial health, social and economic advantages for SBRT and is driving increasing use of SBRT. However, evidence is emerging that SBRT accentuates the role of hypoxia in radioresistance [52,53,54,55] because the opportunity for reoxygenation is reduced [56,57], and thus, the impact of a radiosensitiser is increased [52,58,59].

We wished to compare our best radiosensitisers **9**, **34** and **38** with etanidazole (**2**) in tumour xenograft models using SBRT-like radiation schedules. We compared the compounds in the HCT116/54C and UT-SCC-74B models using a single 10 Gy radiation dose as well as a hypo-fractionated 3 × 5 Gy schedule in the HCT116/54C model. We evaluated drug toxicity as bodyweight loss and observed that the radiosensitisers were well tolerated compared to radiation alone and produced no overt clinical signs of toxicity. Etanidazole (**2**) and **34** appeared to provide additional tumour control compared to radiation in the HCT116/54C and UT-SCC-74B 10 Gy single dose models, but these did not translate to significantly prolonged survival times. In the fractionated 5 Gy (qw × 3) dose model in HCT116/54C tumours **2**, **34** and **38,** all significantly increased survival compared to radiation alone, indicating that both **34** and **38** can effectively sensitise radiation to a similar degree to **2** in SBRT-like schedules. The greater activity observed with the fractionated schedule of **34** and **38**, combined with their high tolerance, suggests the use of formulation strategies to enable higher doses or combination with repeat radiation schedules could be used to further enhance efficacy.

In summary, we have identified two new nitroimidazole sulfonamide radiosensitisers **34** and **38** with comparable activity to **2**, and with potential to be used in combination with SBRT. This discovery complements our earlier identification of **9** as an effective radiosensitiser of HCT116 tumour xenografts. We are mindful that further evaluation, including optimised drug and radiation scheduling, with additional pharmacokinetic, efficacy and toxicity testing in both sexes, will be required to complete our preclinical evaluation of these promising lead compounds.

## 4. Materials and Methods

### 4.1. Compounds

#### 4.1.1. General Information

The full chemical names of compounds tested in this study are provided in Table S1. DMF, DCM, MeCN and THF were purchased predried and stored over molecular sieves from Acros Organics. All other reaction solvents were analytical grade. Nonaqueous reactions were carried out under a N_2_ atmosphere, unless otherwise noted. Commercial reagents were used without purification. Flash column chromatography was carried out on a silica gel solid phase (Merck (Darmstadt, Germany) 230–400 mesh) using distilled laboratory grade solvents. Thin layer chromatography was carried out using Merck 60 F254 aluminium plates, precoated with silica. Compounds were identified using UV fluorescence or iodine on silica gel. Analyses were carried out in the Microchemical Laboratory, University of Otago, Dunedin, New Zealand. Melting points were determined on an Electrothermal 2300 Melting Point Apparatus (ThermoFisher Scientific, Waltham, MA, USA). High-resolution mass spectra (HRMS) were measured on an Agilent Technologies (Santa Clara, CA, USA) 6530 Accurate-Mass Quadrupole Time of Flight (Q-TOF) LC/MS interfaced with an Agilent Jet Stream Electrospray Ionisation (ESI) source, allowing positive or negative ions detection. Low-resolution mass spectra were gathered by direct injection of methanolic solutions into an Agilent 6120 mass spectrometer using atmospheric pressure chemical ionization (APCI) mode with a fragmentor voltage of 50 V and a drying gas temperature of 250 °C. NMR spectra were recorded on a Bruker (Billerica, MA, USA) Avance 400 spectrometer (1H nuclei, 400 MHz; 13C nuclei, 100 MHz) in (CD_3_)_2_SO unless specified. All chemical shift (δ) values are reported in parts per million (ppm) relative to the residual ^1^H resonance from the deuterated solvent; coupling constants are reported in Hertz (Hz). Final products were analysed by reverse-phase HPLC (Agilent Zorbax Eclipse XDB C8 5 µm column, 150 mm × 4.6 mm or Alltech Altima C8 5 µm column, 150 mm × 2.1) using an Agilent HP1100 equipped with a photodiode array detector. Mobile phases were gradients of 80% CH_3_CN/20% H_2_O (*v*/*v*) in 45 mM ammonium formate at pH 3.5 and 0.5–1.0 mL/min. Purity was determined by monitoring at 330 ± 50 nm; all key compounds were greater than 95% pure.

#### 4.1.2. Compound Synthesis

Synthesis of *N*-(2,3-Dihydroxypropyl)-1-(2-nitro-1*H*-imidazol-1-yl)methanesulfonamide (**13**) (Scheme 1).

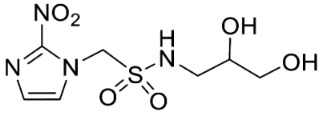

1-Chloro-*N*-((2,2-dimethyl-1,3-dioxolan-4-yl)methyl)methanesulfonamide (**18**).

A solution of chloromethylsulfonyl chloride (3.18 mL, 35.8 mmol) in DCM (5 mL) was added dropwise to a stirred solution of (2,2-dimethyl-1,3-dioxolan-4-yl)methanamine (4.93 g, 37.6 mmol) and iPr_2_NEt (7.7 mL, 46.5 mmol) in dry DCM (100 mL) at 0 °C, and the mixture was stirred at 20 °C for 48 h. The mixture was diluted with DCM (200 mL), washed with water (2 × 100 mL), washed with brine (50 mL), and dried. The solvent was evaporated and the residue purified by chromatography, eluting with a gradient (20–40%) of EtOAc/pet. ether, to give chloride 18 (7.02 g, 81%) as a clear oil: ^1^H NMR (CDCl_3_) δ 4.95 (br s, 1 H, SO_2_NH), 4.75 (d, *J* = 12.2 Hz, 1 H, H-1), 4.28 (d, *J* = 12.2 Hz, 1 H, H-1), 4.28 (ddd, *J* = 12.5, 6.4, 3.5 Hz, 1 H, H-4″), 4.08 (dd, *J* = 8.6, 6.4 Hz, 1 H, H-5″), 3.75 (dd, *J* = 8.6, 5.6 Hz, 1 H, H-5″), 3.39–3.45 (m, 1 H, H-1′), 3.23–3.45 (m, 1 H, H-1′), 1.45 (s, 3 H, 2″-CH_3_), 1.36 (s, 3 H, 2″-CH_3_); *m**/**z* 244.1 (MH^+^, 100%), 246.1 (MH^+^, 40%); HRMS calcd for C_7_H_14_^35^ClNNaO_4_S (M + Na^+^) 266.0224. Found: 266.0234 (−3.6 ppm).

*N*-((2,2-Dimethyl-1,3-dioxolan-4-yl)methyl)-1-(2-nitro-1*H*-imidazol-1-yl)methanesulfonamide (**19**).

A mixture of 2-nitroimidazole (0.89 g, 7.9 mmol), chloride 18 (1.28 g, 5.3 mmol) and Cs_2_CO_3_ (1.80 g, 5.5 mmol) and KI (0.83 g, 5.5 mmol) in DMF (20 mL) was stirred at 70 °C for 6 h. The solvent was evaporated and the residue partitioned between EtOAc (150 mL) and water (50 mL). The organic fraction was washed with water (2 × 50 mL), washed with brine (50 mL), dried, and the solvent evaporated. The residue was purified by column chromatography, eluting with a gradient (50–100%) of EtOAc/pet. ether, to give acetonide 19 (0.71 g, 42%) as a white powder: mp 130–131 °C; ^1^H NMR (CDCl_3_) δ 7.37 (d, *J* = 1.2 Hz, 1 H, H-5), 7.15 (d, *J* = 1.2 Hz, 1 H, H-4), 5.80 (d, *J* = 14.1 Hz, 1 H, H-1′), 5.68–5.73 (m, 2 H, H-1′, SO_2_NH), 4.18–4.25 (m, 1 H, H-4′′′), 4.06 (dd, *J* = 8.7, 6.5 Hz, 1 H, H-5′′′), 3.69 (dd, *J* = 8.7, 5.6 Hz, 1 H, H-5′′′), 3.17–3.19 (m, 2 H, H-1″), 1.43 (s, 3 H, 2′′′-CH_3_), 1.35 (s, 3 H, 2′′′-CH_3_); MS *m*/*z* 321.3 (MH^+^, 100%). Analysis calcd for C_10_H_16_N_4_O_6_S: C, 37.50; H, 5.03; N, 17.49. Found: C, 37.15; H, 4.99; N, 17.51%.

*N*-(2,3-Dihydroxypropyl)-1-(2-nitro-1*H*-imidazol-1-yl)methanesulfonamide (**13**).

A solution of acetonide 19 (483 mg, 1.5 mmol) was stirred in a mixture of HOAc/water/THF (3:1:1, 35 mL) at 50 °C for 24 h. The solvent was evaporated and the residue crystallised from MeOH/iPr_2_O to give diol 13 (389 mg, 92%) as a white powder: mp 127–129 °C; ^1^H NMR δ 7.73 (br s, 1 H, SO_2_NH), 7.70 (d, *J* = 1.2 Hz, 1 H, H-5′), 7.26 (d, *J* = 1.2 Hz, 1 H, H-4′), 5.87 (s, 1 H, H-1), 4.95 (d, *J* = 5.2 Hz, 1 H, 2″-OH), 4.60 (t, *J* = 5.6 Hz, 1 H, 3″-OH), 3.46–3.53 (m, 1 H, H-2″), 3.26–3.34 (m, 2 H, H-3″), 3.08 (br d, *J* = 13.2 Hz, 1 H, H-1″), 2.90 (br dd, *J* = 13.2, 7.1 Hz, 1 H, H-1″); ^13^C NMR δ 144.4, 128.2, 127.8, 70.7, 63.3, 62.6, 45.8; MS *m*/*z* 280.1 (MH^+^, 100%). Analysis calcd for C_7_H_12_N_4_O_6_S: C, 30.00; H, 4.32; N, 19.99. Found: C, 30.05; H, 4.30; N, 19.72%. HPLC purity 99.6% (Supplementary File)

Synthesis of 1-(2-Nitro-1*H*-imidazol-1-yl)-*N*-(1,3,4-trihydroxybutan-2-yl)methanesulfonamide (**14**) (Scheme 1).

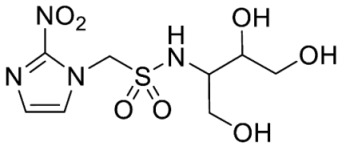

*cis*-2-Butene-1,4-di(tert-butyldimethylsilyl) ether (**20**) [37].

A solution of TBDMSCl (17.52 g, 116.2 mmol) in dry DCM (50 mL) was added dropwise to a stirred solution of *cis*-2-butene-1,4-diol (5.00 g, 56.7 mmol), imidazole (7.73 g, 113.5 mmol) and DMAP (346 mg, 2.8 mmol) in dry DCM (200 mL) at 5 °C, and the mixture was stirred at 20 °C for 16 h. The mixture was diluted with DCM (200 mL) and washed with water (3 × 100 mL), washed with brine (100 mL), dried, and the solvent evaporated. The residue was purified by column chromatography, eluting with 5% EtOAc/pet. ether, to give silyl ether 20 (17.40 g, 97%) as a colourless oil: MS *m*/*z* 317.3 (MH^+^, 100%), which was used directly.

2,3-Bis(((*tert*-butyldimethylsilyl)oxy)methyl)oxirane (**21**) [37].

MCPBA (12.33 g, 71.44 mmol) was added in portions to a stirred solution of silyl ether 20 (17.40 g, 54.95 mmol) in dry DCM (150 mL) at 5 °C, and the mixture was stirred at 20 °C for 16 h. The mixture was diluted with DCM (150 mL) and washed sequentially with sat. aq. sodium bisulfite (150 mL), then, sat. aq. NaHCO_3_ solution (3 × 100 mL). The organic fraction was dried, and the solvent was evaporated. The residue was purified by column chromatography, eluting with 5% EtOAc/pet ether, to give oxirane 21 (17.58 g, 96%) as a colourless oil: ^1^H NMR (CDCl_3_) δ 3.81 (dd, *J* = 11.8, 4.2 Hz, 2 H, H-1, H-4), 3.72 (ddd, *J* = 11.8, 4.6, 1.6 Hz, 2 H, H-1, H-4), 3.11–3.16 (m, 2 H, H-2, H-3), 0.91 [s, 18 H, 2 × OSiC(CH_3_)_3_], 0.09 [2 s, 12 H, 2 × OSi(CH_3_)_2_]; MS *m*/*z* 333.3 (MH^+^, 100%).

2-Azido 1,3,4-tri-((*tert*-butyldimethylsilyl)oxy)butane (**22**).

A mixture of oxirane 21 (17.58 g, 52.84 mmol), NaN_3_ (12.03 g, 185.0 mmol) and NH_4_Cl (9.90 g, 185.0 mmol) in dry DMF (150 mL) was stirred at 110 °C for 5 h. The mixture was diluted with EtOAc (300 mL), and the mixture was washed with water 4 × 200 mL). The organic fraction was dried, and the solvent evaporated. The residue was purified by column chromatography, eluting with a gradient (5–40%) of EtOAc/pet. ether, to give (i) azido trisilyl ether 22 (6.45 g, 25%) as a colourless oil: ^1^H NMR (CDCl_3_) δ 3.80–3.85 (m, 3 H, H-1, H-4), 3.65 (dd, *J* = 9.9, 7.7 Hz, 1 H, H-4), 3.54 (dd, *J* = 9.9, 5.1 Hz, 1 H, H-3), 3.40 (ddd, *J* = 6.9, 6.6, 2.7 Hz, 1 H, H-2), 0.91 [s, 9 H, OSiC(CH_3_)_3_], 0.90 [s, 9 H, OSiC(CH_3_)_3_], 0.89 [s, 9 H, OSiC(CH_3_)_3_], 0.09 [s, 6 H, OSi(CH_3_)_2_], 0.07 [s, 6 H, OSi(CH_3_)_2_], 0.06 [2 × s, 6 H, 3-OSi(CH_3_)_2_]; MS *m*/*z* 489.4 (MH^+^, 100%); and a mixture of alcohols (23, 24) from which 2-azido-(1,3-di-((*tert*-butyldimethylsilyl)oxy)butan-4-ol (**23**) was isolated (4.53 g, 23%) as a colourless oil: ^1^H NMR (CDCl_3_) δ 3.94 (dd, *J* = 10.6, 4.4 Hz, 1 H, H-1), 3.87 (dd, *J* = 10.6, 6.4 Hz, 1 H, H-1), 3.76–3.81 (m, 1 H, H-3), 3.64–3.66 (m, 2 H, H-4), 3.50–3.55 (m, 1 H, H-2), 0.92 [s, 9 H, OSiC(CH_3_)_3_], 0.90 [s, 9 H, OSiC(CH_3_)_3_], 0.11 [s, 6 H, 1-OSi(CH_3_)_2_], 0.08 [2 × s, 6 H, 3-OSi(CH_3_)_2_]; MS *m/z* 376.4 (MH^+^, 100%).

1,3,4-Tri-((*tert*-butyldimethylsilyl)oxy)butan-2-amine (**25**).

A mixture of azide 22 (1.30 g, 2.65 mmol) and Pd/C (5%, 50 mg) in EtOAc/EtOH (1:1, 60 mL) was stirred under an atmosphere of H_2_ (50 psi) for 4 h. The mixture was filtered through diatomaceous earth, the pad washed with EtOAc (10 mL), and the solvent evaporated to give amine 25 (1.228 g, 100%) as a colourless oil: ^1^H NMR (CDCl_3_) δ 3.78 (ddd, *J* = 5.4, 2.4, 2.0 Hz, 1 H, H-3), 3.65 (dd, *J* = 10.0, 7.2 Hz, 1 H, H-1), 3.46–3.56 (m, 3 H, H-1, H-4), 2.90 (dt, *J* = 6.9, 2.3 Hz, 1 H, H-2), 2.70 (br s, 2 H, NH_2_), 0.90 [s, 9 H, OSiC(CH_3_)_3_], 0.89 [s, 9 H, OSiC(CH_3_)_3_], 0.88 [s, 9 H, OSiC(CH_3_)_3_], 0.07 [2 × s, 6 H, 3-OSi(CH_3_)_2_], 0.05 [s, 12 H, 2 × OSi(CH_3_)_2_]; MS *m*/*z* 464.3 (MH^+^, 100%).

1-Bromo-N-(1,3,4-tri-((tert-butyldimethylsilyl)oxy)butan-2-yl)methanesulfonamide (**26**).

A solution of bromomethanesulfonyl chloride (0.51 g, 2.65 mmol) in DCM (3 mL) was added dropwise to a stirred solution of amine 25 (1.228 g, 2.65 mmol) and iPr_2_NEt (0.71 mL, 0.51 mmol) in dry DCM (30 mL) at 5 °C, and the mixture was stirred at 20 °C for 16 h. The mixture was diluted with DCM (150 mL) and washed with water (4 × 50 mL), washed with brine (50 mL), dried, and the solvent evaporated. The residue was purified by column chromatography, eluting with a gradient (2–10%) of EtOAc/pet. ether, to give bromide 26 (0.894 g, 54%) as a colourless oil: ^1^H NMR (CDCl_3_) δ 4.97 (d, *J* = 8.6 Hz, 1 H, SO_2_NH), 4.57 (d, *J* = 11.3 Hz, 1 H, H-1). 4.50 (d, *J* = 11.3 Hz, 1 H, H-1), 4.02 (dd, *J* = 9.2, 5.9, 1.2 Hz, 1 H, H-3′), 3.74 (dd, *J* = 8.8, 5.1 Hz, 1 H, H-2′), 3.55–3.68 (m, 4 H, H-1′, H-4′), 0.90 [s, 9 H, OSiC(CH_3_)_3_], 0.89 [s, 9 H, OSiC(CH_3_)_3_], 0.88 [s, 9 H, OSiC(CH_3_)_3_], 0.08 [2 × s, 6 H, 3-OSi(CH_3_)_2_], 0.07 [s, 12 H, 2 × OSi(CH_3_)_2_]; MS *m*/*z* 620.3 (MH^+^, 100%), 622.3 (MH^+^, 100%).

1-(2-Nitro-1*H*-imidazol-1-yl)-*N*-(1,3,4-tri-((*tert*-butyldimethylsilyl)oxy)butan-2-yl)methanesulfonamide (**27**).

A mixture of 2-nitroimidazole (195 mg, 1.72 mmol), bromide 26 (894 mg, 1.44 mmol), NaI (216 mg, 1.44 mmol) and Cs_2_CO_3_ (560 mg, 1.72 mmol) in dry DMF (25 mL) was stirred at 80 °C for 4 h. The mixture was cooled to 20 °C and diluted with EtOAc (100 mL) and washed with water (4 × 100 mL), washed with brine (50 mL), dried, and the solvent evaporated. The residue was purified by column chromatography, eluting with a gradient (20–40%) of EtOAc/pet. ether, to give sulfonamide 27 (0.894 g, 54%) as a colourless oil: ^1^H NMR (CDCl_3_) δ 7.36 (d, *J* = 1.2 Hz, 1 H, H-5″), 7.19 (d, *J* = 1.2 Hz, 1 H, H-4″), 5.93 (d, *J* = 13.9 Hz, 1 H, H-1), 5.71 (d, *J* = 13.9 Hz, 1 H, H-1), 5.02 (d, *J* = 8.6 Hz, 1 H, SO_2_NH), 3.95 (ddd, *J* = 9.2, 5.3, 1.7 Hz, 1 H, H-3′), 3.68 (m, 1 H, H-2′), 3.60–3.65 (m, 2 H, H-4′), 3.57 (dd, *J* = 10.3, 5.3 Hz, 1 H, H-1′), 3.48 (dd, *J* = 10.3, 5.3 Hz, 1 H, H-1′), 0.92 [s, 9 H, OSiC(CH_3_)_3_], 0.88 [s, 9 H, OSiC(CH_3_)_3_], 0.87 [s, 9 H, OSiC(CH_3_)_3_], 0.11 [s, 6 H, OSi(CH_3_)_2_], 0.08 [2 × s, 6 H, 3-OSi(CH_3_)_2_], 0.07 [s, 6 H, OSi(CH_3_)_2_]; MS *m*/*z* 650.9 (M-H, 100%).

1-(2-Nitro-1*H*-imidazol-1-yl)-N-(1,3,4-trihydroxybutan-2-yl)methanesulfonamide (**14**).

A solution of TBAF (1.0 M, 0.15 mL, 0.15 mmol) was added to a solution of sulfonamide 27 (84 mg, 0.13 mmol) in THF (6 mL) at 20 °C, and the solution was stirred for 24 h. The residue was purified by column chromatography, eluting with a gradient (10–20%) of MeOH/DCM, to give sulfonamide 14 (24 mg, 61%) as a white solid: m.p. (EtOAc) 128–131 °C; ^1^H NMR δ 7.75 (d, *J* = 1.1 Hz, 1 H, H-5″), 7.59 (br s, 1 H, SO_2_NH), 7.25 (d, *J* = 1.1 Hz, 1 H, H-4″), 5.90 (d, *J* = 14.1 Hz, 1 H, H-1), 5.84 (d, *J* = 14.1 Hz, 1 H, H-1), 5.00 (br t, *J* = 4.8 Hz, 1 H, 3-OH), 4.84 (br s, 1 H, OH), 4.58 (br s, 1 H, OH), 3.52–3.58 (m, 2 H, H-1′, H-3′), 3.37–3.46 (m, 4 H, H-1′, H-2′, H-4′); ^13^C NMR δ 144.5, 128.0, 127.7, 70.7, 63.6, 62.4, 61.5, 58.0; HRMS calcd for C_8_H_15_N_4_O_7_S (M + H^+^) m/z 311.0656. Found: 311.0651 (−1.69 ppm). HPLC purity 98.9% (Supplementary File).

Synthesis of *N*-(2,3-Dihydroxypropyl)-2-(2-nitro-1*H*-imidazol-1-yl)ethane-1-sulfonamide (**15**) (Scheme 2).

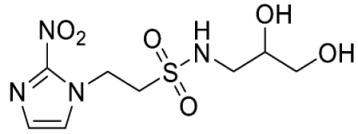

*N*-((2,2-Dimethyl-1,3-dioxolan-4-yl)methyl)-2-(2-nitro-1*H*-imidazol-1-yl)ethane-1-sulfonamide (**29**).

A solution of *S*-(2-(2-nitro-1*H*-imidazol-1-yl)ethyl) ethanethioate 28 [34] (1.03 g, 4.79 mmol) in CH_3_CN (8 mL) was added dropwise to a mixture of NCS (2.56 g, 19.14 mmol) in aqueous HCl (2 M, 2 mL) and CH_3_CN (6 mL) at 10 °C. The mixture was stirred at 10–15 °C for 30 min, and allowed to warm to 20 °C over 10 min. The mixture was partitioned between EtOAc (150 mL) and aq. NaHCO_3_ (50 mL). The organic phase was washed with dilute NaCl (3 × 50 mL), dried, and the solvent was evaporated to give the crude sulfonyl chloride which was used without further purification. iPr_2_NEt (1.28 mL, 7.19 mmol) and (2,2-dimethyl-1,3-dioxolan-4-yl)methanamine (0.75 mL, 5.75 mmol) were successively added to a suspension of the crude sulfonyl chloride in anhydrous DCM (10 mL) at 5 °C. The mixture was allowed to warm to 20 °C and stirred for 16 h. The mixture was diluted with DCM (100 mL), washed with water (3 × 50 mL), washed with brine (50 mL), dried, and the solvent was evaporated. The residue was purified by column chromatography, eluting with 80% EtOAc/pet. ether, to give sulfonamide 29 (382 mg, 24%) as a colourless oil: ^1^H NMR (CDCl_3_) δ 7.25 (d, *J* = 1.0 Hz, 1 H, H-5′), 7.19 (d, *J* = 1.0 Hz, 1 H, H-4′), 4.78–4.92 (m, 2 H, H-2), 4.63 (br t, *J* = 6.0 Hz, 1 H, SO_2_NH), 4.13–4.20 (m, 1 H, H-4′′′), 4.05 (dd, *J* = 8.6, 6.5 Hz, 1 H, H-5′′′), 3.65–3.74 (m, 2 H, H-1′, H-5′′′), 3.60 (dt, *J* = 14.5, 6.3 Hz, 1 H, H-1), 3.27 (ddd, *J* = 13.6, 6.6, 3.4 Hz, 1 H, H-1″), 3.17 (ddd, *J* = 13.6, 7.4, 5.7 Hz, 1 H, H-1″), 1.43 (s, 3 H, 2″′-CH_3_), 1.33 (s, 3 H, 2′′′-CH_3_); HRMS calcd for C_11_H_18_N_4_O_6_S (M^+^) *m*/*z* 334.0974. Found: 334.0964 (−5.0 ppm).

*N*-(2,3-Dihydroxypropyl)-2-(2-nitro-1*H*-imidazol-1-yl)ethane-1-sulfonamide (**15**).

A solution of nitroimidazole 29 (0.38 g, 1.14 mmol) in a mixture of HOAc/THF/H_2_O (3:1:1, 25 mL) was stirred at 50 °C for 16 h. The solvents were evaporated and the residue was triturated with pet. ether to give sulfonamide 15 (285 mg, 85%) as white crystals: mp (pet. ether) 120–123 °C; ^1^H NMR δ 7.69 (d, *J* = 1.0 Hz, 1 H, H-5′), 7.28 (br s, 1 H, SO_2_NH), 7.17 (d, *J* = 1.0 Hz, 1 H, H-4′), 4.87 (d, *J* = 5.1 Hz, 1 H, 2″-OH), 4.74 (dd, *J* = 7.2, 6.7 Hz, 2 H, H-2), 4.58 (br t, *J* = 4.8 Hz, 1 H, 3″-OH), 3.62 (dd, *J* = 7.2, 6.7 Hz, 2 H, H-1), 3.47–3.55 (m, 1 H, H-2″), 3.25–3.34 (m, 2 H, H-3″), 3.09 (dd, *J* = 13.4, 4.2 Hz, 1 H, H-1″), 2.89 (dd, *J* = 13.4, 7.2 Hz, 1 H, H-1″); ^13^C NMR δ 144.6, 128.2, 127.6, 70.7, 63.4, 50.6, 45.7, 44.1. Analysis calcd for C_8_H_14_N_4_O_6_S: C, 32.65; H, 4.80; N, 19.04. Found: C, 33.00; H, 4.83; N, 19.02%. HPLC purity 99.3% (Supplementary File).

Synthesis of *N*-(2-Morpholinoethyl)-2-(5-nitro-1*H*-imidazol-1-yl)ethane-1-sulfonamide (**16**) (Scheme 2).

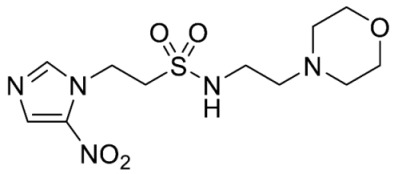


A solution of nitroimidazole thioester 30 [34] (1.43 g, 6.67 mmol) in 1M HCl/MeCN (1:5, 36 mL) was cooled to 10 °C. NCS (3.56 g, 16.67 mmol) was added in portions and the resulting mixture was allowed to warm to 20 °C over 1 h. The resulting mixture was diluted with EtOAc (50 mL) and washed with brine (2 × 50 mL). The organic layer was dried and concentrated to ~20 mL. The crude sulfonyl chloride solution was added slowly (over 1.5 h) to a stirred mixture of 4-(2-aminoethyl)morpholine (0.87 mL, 6.67 mmol) and iPr_2_NEt (3.3 mL, 18.66 mmol) in EtOAc (10 mL) at 0 °C. The reaction mixture was allowed to warm to 20 °C over 3.5 h. The resulting mixture was washed with water (50 mL), washed with brine (50 mL), dried, and the solvent evaporated. The residue was purified by column chromatography, eluting with a gradient (4–5%) of MeOH/DCM, to give amine (155 mg, 7%). The free base (129 mg, 0.39 mmol) was treated with 2M HCl/diethyl ether (0.195 mL, 0.39 mmol) and removed by filtration to obtain 16 (121 mg, 85%) as an off-white solid: m.p. (diethyl ether) 162–166 °C; ^1^H NMR (CDCl_3_) δ 11.01 (br s, 1 H, HCl), 8.21 (d, *J* = 1.1 Hz, 1 H, H-2), 8.11 (d, *J* = 1.1 Hz, 1 H, H-4), 7.95 (t, *J* = 6.0 Hz, 1 H, NH), 4.75 (t, *J* = 6.8 Hz, 2 H, NCH_2_), 3.96 (m, 2 H, H_2_-3′ or H_2_-5′), 3.80 (t, *J* = 11.7 Hz, 2 H, H_2_-3′ or H_2_-5′), 3.68 (t, *J* = 6.8 Hz, 2 H, CH_2_SO_2_), 3.43 (m, 4 H, H_2_-2′ or H_2_-6′), 3.24 (m, 2 H, CH_2_N), 3.11 (m, 2 H, H_2_-2′ or H_2_-6′); ^13^C NMR (CDCl_3_) δ 143.7, 138.3, 133.4, 63.0 (2), 55.6, 51.2 (2), 50.1, 41.7, 36.5; MS 334.2 (MH^+^, 100%); HRESIMS calcd for C_11_H_20_N_5_O_5_S: 334.11776. Found: 334.11797. HPLC purity 99.8% (Supplementary File).

Synthesis of 2-Hydroxy-*N*-(2-(2-nitro-1*H*-imidazol-1-yl)ethyl)ethane-1-sulfonamide (**17**) (Scheme 2).

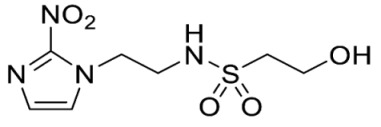

*tert*-Butyl (2-(2-nitro-1*H*-imidazol-1-yl)ethyl)carbamate (**31**).

Cs_2_CO_3_ (3.83 g, 11.75 mmol) was added to a solution of 2-nitroimidazole (1.90 mg, 16.78 mmol), *tert*-butyl (2-bromoethyl)carbamate (2.51 g, 11.19 mmol) and NaI (1.76 mg, 11.75 mmol) in DMF (30 mL). The reaction mixture was heated at 80 °C for 24 h. DMF was evaporated, and the residue diluted with EtOAc (50 mL). The organic layer was washed with water (2 × 50 mL), washed with brine (50 mL), dried, and the solvent evaporated. The residue was purified by column chromatography, eluting with EtOAc, to give carbamate 31 (1.92 g, 67%) as a pale yellow solid: m.p. (EtOAc) 112–114 °C; ^1^H NMR (CDCl_3_) δ 7.16 (d, *J* = 0.8 Hz, 1 H, H-5′), 7.07 (s, 1 H, H-4′), 4.70 (br s, 1 H, NH), 4.58 (t, *J* = 5.8 Hz, 2 H, NCH_2_), 3.56 (dt, *J* = 5.8, 6.1 Hz, 2 H, CH_2_NH), 1.42 (s, 9H, *t*Bu); MS 257.2 (MH^+^, 100%).

2-(2-Nitro-1*H*-imidazol-1-yl)ethan-1-amine (**32**).

Carbamate 31 (1.92 g, 7.48 mmol) was stirred in TFA (5 mL) for 1 h. The reaction mixture was diluted with EtOAc (30 mL), and the resulting white solid was filtered to give amine 32 as the trifluoroacetate salt (1.85 g, 98%) as a white solid: m.p. (EtOAc) 125–128 °C; ^1^H NMR δ 7.83 (br s, 2 H, NH_2_), 7.63 (d, *J* = 1.1 Hz, 1 H, H-5′), 7.23 (d, *J* = 1.1 Hz, 1 H, H-4′), 4.61 (t, *J* = 6.1 Hz, 2 H, NCH_2_), 3.30 (obscured t, *J* = 6.1 Hz, 2 H, CH_2_NH_2_); MS 157.1 (MH^+^, 100%).

2-Hydroxy-N-(2-(2-nitro-1*H*-imidazol-1-yl)ethyl)ethane-1-sulfonamide (**17**).

NCS (6.47 g, 48.5 mmol) was added over 1 min to a stirred solution of mercaptoethanol (1.13 mL, 16.1 mmol), tetrabutylammonium chloride (10.14 g, 36.5 mmol) and water (0.72 mL, 40.4 mmol) in MeCN (10 mL) at 0 °C. After 2 hours, amine 32 (2.05 g, 8.1 mmol) and iPr_2_NEt (7.05 mL, 40.4 mmol) in MeCN (10 mL) was added. The resulting mixture was allowed to stir at 20 °C for 19 h. The reaction mixture was concentrated under reduced pressure and diluted in EtOAc. The filtrate was evaporated and purified by column chromatography, eluting with a gradient (1–2%) of MeOH/EtOAc, to give 17 (470 mg, 22%) as a white solid: m.p. (DCM) 124–127 °C; ^1^H NMR (CDCl_3_) δ 7.59 (d, *J* = 1.0 Hz, 1 H, H-5), 7.19 (d, *J* = 1.0 Hz, 1 H, H-4), 7.18 (br s, 1 H, NH), 4.91 (m, 1 H, OH), 4.46 (t, *J* = 6.1 Hz, 2 H, NCH_2_), 3.69 (td, *J* = 6.3, 4.7 Hz, 2 H, CH_2_OH), 3.39 (t, *J* = 6.1 Hz, 2 H, CH_2_NH), 3.15 (t, *J* = 6.3 Hz, 2 H, SO_2_CH_2_); ^13^C NMR (CDCl_3_) δ 144.7, 128.5, 127.7, 55.6, 53.9, 49.6, 41.8; MS 265.1 (MH^+^, 100%); HRESIMS calcd for C_7_H_13_N_4_O_5_S (M + H^+^) *m*/*z* 264.0523. Found: 264.0525 (0.61 ppm). HPLC purity 98.4% (Supplementary File).

### 4.2. Assays

#### 4.2.1. Solubility and Stability

The solubility of the final products was determined by HPLC analysis after incubation of saturated solutions of the drug in αMEM containing 5% foetal calf serum (FCS) and 1% DMSO at 37 °C to mimic cell culture conditions. The stability of the same solutions after 24 h incubation at 37 °C was measured by HPLC analysis.

#### 4.2.2. In Vitro Cytotoxicity Testing

HCT116/54C is an HCT116 subline that originated from a mixed culture with UT-SCC-54C cells [60], which was supplied alongside UT-SCC-74B by Prof. Bradly Wouters (University Health Network, Toronto, ON, Canada). HCT116/54C and UT-SCC-74B cells were passaged in minimum essential media (MEM, ThermoFisher Scientific, Waltham, MA, USA) with 10% foetal calf serum (FCS, Moregate Biotech, Hamilton, New Zealand), 4.5 mg/mL D-glucose (Sigma-Aldrich, St. Louis, MO, USA) and 20 mM HEPES (Sigma-Aldrich). FaDu cells were sourced from American Type Culture Collection and cultured in αMEM (ThermoFisher Scientific) with 10% FCS. All cell lines were confirmed mycoplasma-free by PlasmoTest (InvivoGen, San Diego, CA, USA) and were derived from STR-authenticated cryopreserved stocks. Log-phase cells were seeded into 96-well plates at 800 cells/well for HCT116/54C and 1500 cells/well for FaDu. Cells were allowed to attach for 2 h, then, test compounds were added by dilution from DMSO stocks to give a top concentration in <1% DMSO before serial 3-fold dilution in the plates. After 4 h, cultures were washed 3 times with fresh medium and grown for a further 5 days before staining with sulforhodamine B to determine IC_50_ values as previously [61]. For hypoxic exposure to compounds, cells were pelleted by centrifugation, transferred to a Pd-catalyst anaerobic chamber (Bactron-II, Shel Lab), resuspended in anoxic medium, and exposed to drugs as above, but using medium and plates that had been equilibrated in the chamber for at least 3 days. After drug washout, cells were grown and stained as for the oxic IC_50_ assays. The hypoxic cytotoxicity ratio HCR was determined as HCR = IC_50(oxic)_/IC_50(anoxic)_.

#### 4.2.3. In Vitro Radiosensitisation Testing

Cells were seeded onto 96-well plates at 10^5^ cells per well and allowed to attach for 2 h. Replicates were treated with compound at the respective anoxic IC_50_ concentration 30 min prior to irradiation. For anoxic irradiation, plates were transferred to a custom-built, air-tight, stainless steel, portable box (13 × 16.5 × 3 cm) within the anaerobic chamber, then, sealed and transported to the radiation machine. The plates were irradiated (Eldorado 78 ^60^Co teletherapy unit, ~2 Gy/min) with 15 Gy under anoxia at room temperature for SR experiments. A metal wedge placed on the top of the metal chamber was used to achieve a graduated radiation dose across the plate, varying from 7 to 29 Gy, calibrated by Fricke dosimetry with ammonium thiocyanate as previously described [62,63] for SER experiments. The control plate (compound alone, no radiation) was left inside the anaerobic chamber at room temperature during the irradiation period. After treatment, the cells were trypsinized and suspended in usual media + 1% penicillin-streptomycin (ThermoFisher Scientific), and 10-fold serial dilutions were plated for clonogenic survival. After 10 days, plates were stained with methylene blue (2 g/L in EtOH/H_2_O, 1:1 *v*/*v*) and colonies with more than 50 cells were counted. The surviving fraction (SF) was determined as: SF = PE_(irradiated)_/PE_(control)_ where the plating efficiency (PE) = (No. of colonies)/(No. of cells plated). SF was plotted against radiation dose. Survival ratios (SR) were calculated: SR = (cell survival with radiation)/(cell survival with drug + radiation). Sensitizer enhancement ratios (SER) were calculated: SER = (radiation dose for 1% survival without compound)/(radiation dose for 1% survival with compound). Misonidazole (**1**) at its hypoxic IC_50_ (0.5 mM) was used as an intra-experiment control.

### 4.3. Animal Experiments

Specific pathogen-free six- to eight-week-old female NIH-III mice, bred in the Vernon Jansen Unit, University of Auckland, were used for animal experiments. Animals had ad libitum access to food and water and were maintained in microisolator cages on a 12 h light/dark cycle. All treatments were administered by i.v. injection at a dose of 2.2 mmol/kg and injection volume of 10 mL/kg. Compounds **2**, **34** and **38** were administered as solutions in saline containing 1% DMSO or for **9**–**12** as solutions in saline containing 2 molar equivalents of NaHCO_3_. Control animals received an i.v. injection of saline at 10 mL/kg. All animal experiments were conducted under the auspices of the University of Auckland Animal Ethics Committee (approval 1781).

#### 4.3.1. Pharmacokinetics

Female NIH-III mice were injected with a single i.v. dose of 34 at its MAD of 2.2 mmol/kg in saline. Blood was collected by cardiac puncture under terminal CO_2_ anaesthesia at 5, 10, 30, 60 and 120 min after dosing. Blood was immediately centrifuged (3000× *g*, 3 min), and plasma was stored at −80 °C for HPLC. Tumour samples were prepared and analysed by HPLC using standard protocols established in our laboratory [63,64,65].

#### 4.3.2. Tumour Radiosensitisation: Ex Vivo Clonogenic Assay

Compounds were evaluated for in vivo radiosensitisation of hypoxic cells in HCT116/54C and UT-SCC-74B human tumour xenografts using clonogenic survival as an endpoint, using previously report methods [66]. Tumours were grown subcutaneously on the flanks of NIH-III mice by injecting 5 × 10^6^ HCT116/54C cells or UT-SCC-74B cells. Once tumours reached a volume of approximately 250 mm^3^, mice were randomly assigned to 4 treatment groups and treated with: (A) vehicle control, (B) 10 Gy RAD only, (C) sensitiser only, or (D) sensitiser given before 10 Gy RAD. Drugs were administered as a single i.v. dose at 2.2 mmol/kg. Mice were injected with drug and irradiated (Eldorado 78 ^60^Co teletherapy unit, ~2 Gy/min, whole body irradiation at 10 Gy) 10 min later. Tumours were excised 18 h after treatment, and tumour cells were dissociated enzymatically and plated to determine the number of surviving (clonogenic) cells per gram of tumour tissue [66]. SF and SR were calculated as described above. Statistical significance of drug effects was tested using one-way ANOVA with Sidak tests for multiple comparisons vs. radiation alone (Table 5).

#### 4.3.3. Tumour Radiosensitisation: Tumour Growth Inhibition

Compounds were evaluated for their ability to inhibit tumour growth in combination with a single dose of radiation (10 Gy) in HCT116/54C or UT-SCC-74B tumour xenografts on the backs of NIH-III mice. In addition, a fractionated radiation schedule (5 Gy on Days 1, 7 and 14) was explored in HCT116/54C tumour xenografts. Mice were randomly assigned to 4 treatment groups and treated with: (A) vehicle control, (B) 10 Gy RAD only, (C) sensitiser only, or (D) sensitiser given 10 min before 10 Gy RAD. Drugs were administered by i.v. injection at 2.2 mmol/kg either as a single dose or three doses on Days 1, 7 and 14 in the fractionated study. Treatment commenced when the average tumour size reached approximately 250 mm^3^, and tumour size was measured until study endpoint. Mice were placed in a custom-built lead-shielded jig that allowed irradiation of the whole tumour but shielded the mouse from radiation. Survival analysis represented the time taken for tumours to increase in relative tumour volume by 4× from the start of treatment (RTV^4^). The significance of differences in activity between the groups was assessed by log-rank with Holm–Sidak multiple comparison analysis.

## Figures and Tables

**Table 1:** Physicochemical properties of new nitroimidazole sulfonamide radiosensitiser compounds.

No.	Structure	Purity %	Solubility ^1^ (mM)	% Stability in αMEM
**13**	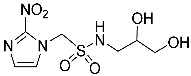	99.6	110	99.3
**14**	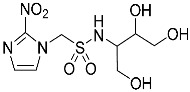	98.9	95.8	98.5
**15**	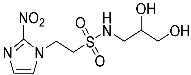	99.3	84.3	99.9
**16**	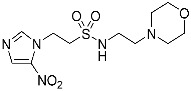	99.8	83.05	99.9
**17**	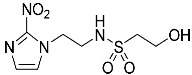	98.4	>101.2	100.0

^1^ Solubility in α-MEM culture medium containing 5% foetal calf serum.

**Table 2:** Cytotoxicity (mean ± SEM, *n* = 3–4) of nitroimidazole sulfonamide radiosensitiser compounds.

No.	Structure	HCT116/54C			FaDu			UT-SCC-74B		
		IC_50_ Oxic (mM)	IC_50_ Anoxic (mM)	HCR	IC_50_ Oxic (mM)	IC_50_ Anoxic (mM)	HCR	IC_50_ Oxic (mM)	IC_50_ Anoxic (mM)	HCR
**1**	1	>25	1.5 ± 0.3	>16.8	>20	1.3 ± 0.2	>15.4	>25	2.1 ± 0.6	>11.9
**2**	2	>25	1.7 ± 0.2	>14.8	>20	1.5 ± 0.3	>13.3	-	-	-
**3**	3	>25	>20	-	>20	13.2 ± 3.4	>1.5	-	-	-
**5**	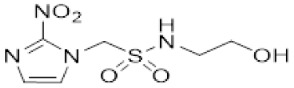	>6.2	0.29 ± 0.02	>21	2.3 ± 0.2	0.23 ± 0.01	10.0	4.3 ± 0.8	0.24 ± 0.04	17.7
**6**	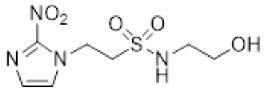	>16	2.2 ± 0.04	>7.3	17.8 ± 2.2	1.2 ± 0.2	14.9	>20	3.0 ± 0.6	>6.6
**7**	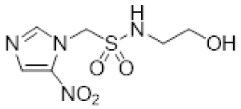	4.6 ± 0.3	1.4 ± 0.1	3.3	5.4 ± 0.7	1.4 ± 0.02	4.0	4.2 ± 0.9	1.3 ± 0.04	3.3
**8**	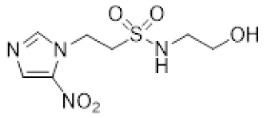	>22	9.0 ± 0.9	>2.4	>24	6.6 ± 0.6	>3.6	>22	10.9 ± 1.3	>2.0
**13**	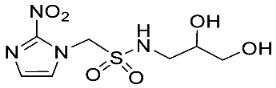	14.5 ± 2.9	4.1 ± 0.1	3.5	8.7 ± 1.1	4.3 ± 0.3	2.0	10.3 ± 1.7	2.6 ± 0.1	4.0
**14**	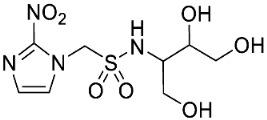	16.4 ± 3.0	12.6 ± 0.5	1.3	5.3 ± 1.1	7.9 ± 1.1	0.67	-	-	-
**15**	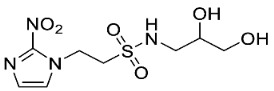	>25	12.8 ± 4.4	>1.9	>25	7.3 ± 0.9	>3.4	-	-	-
**16**	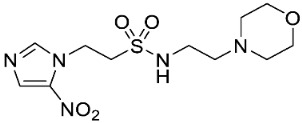	12.8 ± 1.5	12.9 ± 0.9	1.0	>16	6.1 ± 0.8	>2.6	14.1 ± 2.1	10.3 ± 2.6	1.4
**17**	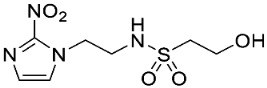	>17	3.2 ± 0.2	>5.3	>18	2.8 ± 0.6	>6.4	>20	2.9 ± 0.5	>6.8
**33**	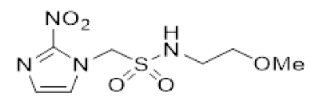	>6.2	0.12 ± 0.002	>51	4.0 ± 1.2	0.09 ± 0.01	46.6	3.7 ± 0.8	0.19 ± 0.02	19.9
**34**	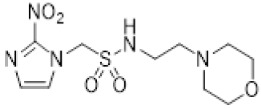	6.4 ± 1.0	0.17 ± 0.01	38.8	3.7 ± 0.1	0.16 ± 0.01	23.3	12.6 ± 1.8	0.51 ± 0.08	24.5
**35**	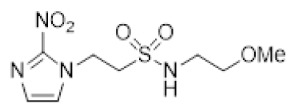	>16	0.97 ± 0.16	>16	16.5 ± 1.6	0.81 ± 0.12	20.4	-	-	-
**36**	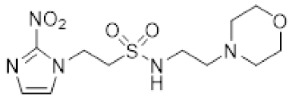	9.2 ± 1.1	0.71 ± 0.13	12.9	7.3 ± 0.6	0.69 ± 0.04	10.6	11.9 ± 1.5	1.6 ± 0.3	7.6
**37**	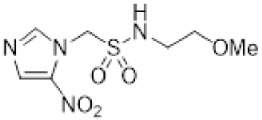	11.4 ± 1.0	4.0 ± 0.7	2.9	12.0 ± 1.9	2.1 ± 0.04	5.7	-	-	-
**38**	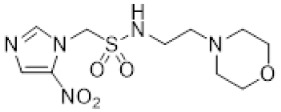	13.7 ± 2.0	1.2 ± 0.1	11.2	10.6 ± 0.6	1.8 ± 0.1	5.8	13.1 ± 2.8	1.5 ± 0.3	8.6
**39**	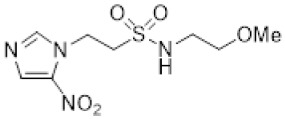	>25	13.0 ± 1.3	>1.9	>19	7.2 ± 0.2	>2.6	-	-	-
**40**	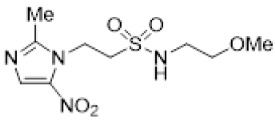	>20	11.0 ± 0.4	>1.8	>22	8.0 ± 0.5	>2.7	-	-	-
**41**	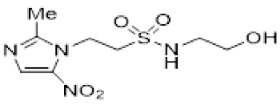	>18	15.5 ± 2.6	>1.1	>25	>22	-	>21	13.7 ± 3.7	>1.5
**42**	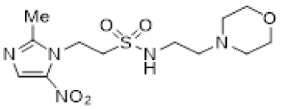	7.6 ± 0.4	4.3 ± 0.2	1.8	9.8 ± 1.2	7.8 ± 0.2	1.3	8.9 ± 1.6	6.6 ± 0.6	1.4
**43**	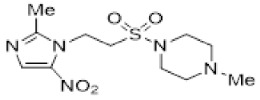	11.7 ± 1.3	9.9 ± 0.3	1.2	13.3 ± 2.5	8.6 ± 0.6	1.6	-	-	-
**44**	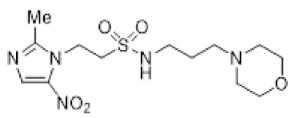	>21	10.3 ± 1.5	>2.0	>16	7.8 ± 0.7	>2.0	13.2 ± 2.0	7.1 ± 1.5	1.9
**45**	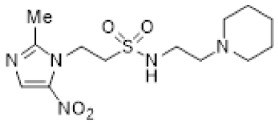	11.8 ± 1.5	2.4 ± 0.4	5.0	4.8 ± 0.3	1.7 ± 0.4	2.8	-	-	-
**46**	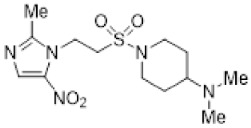	16.9 ± 1.4	2.0 ± 0.3	8.6	9.5 ± 2.0	1.3 ± 0.1	7.3	8.1 ± 0.9	1.9 ± 0.2	4.4

**Table 3:** Sensitiser enhancement ratios in HCT116/54C cells.

Compound		SER ^1^ at 1 mM
Misonidazole **1**		1.46 ± 0.01
Etanidazole **2**		1.18 ± 0.00
Nimorazole **3**		1.20 ± 0.01
**5**	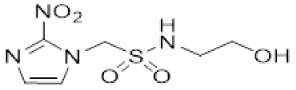	1.72 ± 0.06
**6**	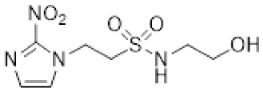	1.39 ± 0.01
**7**	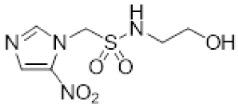	1.41 ± 0.02
**8**	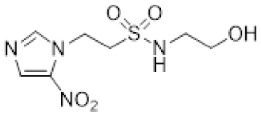	1.27 ± 0.00
**13**	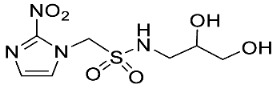	1.00 ± 0.00
**14**	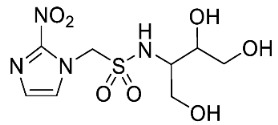	1.00 ± 0.00
**16**	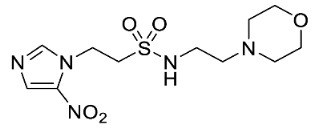	1.31 ± 0.01
**17**	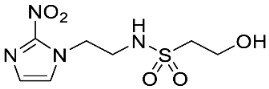	1.35 ± 0.02
**34**	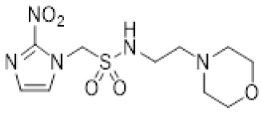	1.92 ± 0.01
**36**	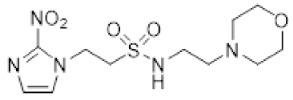	1.57 ± 0.00
**38**	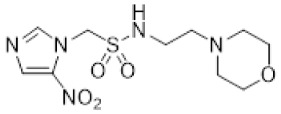	1.39 ± 0.01
**41**	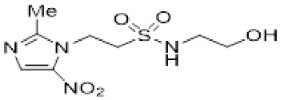	1.16 ± 0.01
**42**	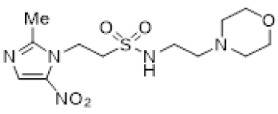	1.16 ± 0.01

^1^ SER, radiation dose for 1% survival/radiation dose for 1% survival with compound. Values are mean ± SEM for *n* = 2 (except **1**, *n* = 14).

**Table 4:** Pharmacokinetic data in NIH-III mice after treatment at 2.2 mmol/kg i.v. injection.

Compound	Plasma Cmax (mM)	T_1/2_ (h)	AUC (mM·h)
**34**	1.53	1.52	0.96
**2** ^1^	3.64	1.05	1.82
**9** ^1^	0.92	2.33	0.55
**10** ^1^	1.14	2.21	0.64
**11** ^1^	1.10	1.72	0.71

^1^ Data from [34].

**Table 5:** In vivo radiosensitisation data.

Compound ^1^	Sensitisation Ratio ^2^	
	HCT116/54C	UT-SCC-74B
**2**	3.34	8.94
**9**	6.22	9.64
**10**	1.78	-
**11**	1.85	-
**12**	3.32	-
**34**	14.9	13.8
**38**	3.43	11.5

^1^ Compounds administered by i.v. injection at 2.2 mmol/kg 5 min prior to 10 Gy radiation. ^2^ In vivo sensitisation ratio = surviving fraction after radiation/surviving fraction after drug + radiation.

## Data Availability

The data presented in this study are available in the article and Supplementary Materials.

## Supplementary Materials

The following supporting information can be downloaded at: https://www.mdpi.com/article/10.3390/molecules28114457/s1, Table S1: Full chemical names of compounds **1**–**46**; Supplementary File: ^1^H, ^13^C and HPLC spectra of compounds **13**–**17**.

## Sample Availability

Samples of the compounds **5**–**46** are available from the authors.

## Abbreviations

AUC, area under the curve; BOC, *tert*-butyloxycarbonyl; C_max_, maximum plasma concentration; CRT, chemoradiotherapy; DCM, dichloromethane; DMF, *N*,*N*-dimethylformamide; DMAP, 4-dimethylaminopyridine; DMSO dimethylsulfoxide; EtOAc, ethyl acetate; EtOH, ethanol; FCS, foetal calf serum; HNSCC, head and neck squamous cell carcinoma; HOAc, acetic acid; iPr_2_O, di-isopropyl ether; MAD, maximum administered dose; MeCN, acetonitrile; MeOH, methanol; MTD, maximum tolerated dose; NCS, *N*-chlorosuccinimide; PE, plating efficiency; RAD, radiation; RT, radiotherapy; SER, sensitiser enhancement ratio; SF, surviving fraction; SR, survival ratio; TBAF, tetrabutylammonium fluoride; TBDMS, *tert*-butyldimethylsilyl; T_½_, drug half-life in plasma; TFA, trifluoroacetic acid; THF, tetrahydrofuran; pet. Ether, petroleum ether boiling range 40–60 °C.
