# Supplementary material for: Design, Synthesis and Anticancer Evaluation of Nitroimidazole Radiosensitisers
Source: Molecules. 2023 May 31;28(11):4457. doi: 10.3390/molecules28114457 (PMC10254852; doi:10.3390/molecules28114457)
Supplement: Supplementary file 1 [file molecules-28-04457-s001.zip › molecules-2409136-supplementary.pdf]

Table S1: Full chemical names of compounds **1** - **46**

1-Methoxy-3-(2-nitro-1*H*-imidazol-1-yl)propan-2-ol (1)
N-(2-Hydroxyethyl)-2-(2-nitro-1*H*-imidazol-1-yl)acetamide (2)
4-(2-(5-Nitro-1*H*-imidazol-1-yl)ethyl)morpholine (3)
3-((2-Nitro-1*H*-imidazol-1-yl)methoxy)butane-1,2,4-triol (4)
N-(2-Hydroxyethyl)(2-nitro-1*H*-imidazol-1-yl)methanesulfonamide (5)
N-(2-Hydroxyethyl)-2-(2-nitro-1*H*-imidazol-1-yl)ethanesulfonamide (6)
N-(2-Hydroxyethyl)(5-nitro-1*H*-imidazol-1-yl)methanesulfonamide (7)
N-(2-Hydroxyethyl)-2-(5-nitro-1*H*-imidazol-1-yl)ethane-1-sulfonamide (8)
2-(((2-Nitro-1*H*-imidazol-1-yl)methyl)sulfonamido)ethyl Dihydrogen Phosphate (9)
2-(((2-Nitro-1*H*-imidazol-1-yl)ethyl)sulfonamido)ethyl Dihydrogen Phosphate (10)
2-(((5-Nitro-1*H*-imidazol-1-yl)methyl)sulfonamido)ethyl Dihydrogen Phosphate (11)
2-((2-(5-Nitro-1*H*-imidazol-1-yl)ethyl)sulfonamido)ethyl Dihydrogen Phosphate (12)
N-(2,3-Dihydroxypropyl)-1-(2-nitro-1*H*-imidazol-1-yl)methanesulfonamide (13)
1-(2-Nitro-1*H*-imidazol-1-yl)-*N*-(1,3,4-trihydroxybutan-2-yl)methanesulfonamide (14)
N-(2,3-Dihydroxypropyl)-2-(2-nitro-1*H*-imidazol-1-yl)ethane-1-sulfonamide (15)
N-(2-Morpholinoethyl)-2-(5-nitro-1*H*-imidazol-1-yl)ethane-1-sulfonamide (16)
2-Hydroxy-*N*-(2-(2-nitro-1*H*-imidazol-1-yl)ethyl)ethane-1-sulfonamide (17)
1-Chloro-*N*-((2,2-dimethyl-1,3-dioxolan-4-yl)methyl)methanesulfonamide (18)
N-((2,2-Dimethyl-1,3-dioxolan-4-yl)methyl)-1-(2-nitro-1*H*-imidazol-1-yl)methanesulfonamide (19)
cis-2-Butene-1,4-di(*tert*-butyldimethylsilyl) ether (20)
2,3-Bis(((*tert*-butyldimethylsilyl)oxy)methyl)oxirane (21)
2-Azido 1,3,4-tri-((*tert*-butyldimethylsilyl)oxy)butane (22)
2-Azido-(1,3-di-((*tert*-butyldimethylsilyl)oxy)butan-4-ol (23)
2-Azido-(1,4-di-((*tert*-butyldimethylsilyl)oxy)butan-3-ol (24)
1,3,4-Tri-((*tert*-butyldimethylsilyl)oxy)butan-2-amine (25)
1-Bromo-*N*-(1,3,4-tri-((*tert*-butyldimethylsilyl)oxy)butan-2-yl)methanesulfonamide (26)
1-(2-Nitro-1*H*-imidazol-1-yl)-*N*-(1,3,4-tri-((*tert*-butyldimethylsilyl)oxy)butan-2-yl)methanesulfonamide (27)
S-(2-(2-Nitro-1*H*-imidazol-1-yl)ethyl) ethanethioate (28)
N-((2,2-Dimethyl-1,3-dioxolan-4-yl)methyl)-2-(2-nitro-1*H*-imidazol-1-yl)ethane-1-sulfonamide (29)
S-(2-(5-Nitro-1*H*-imidazol-1-yl)ethyl) ethanethioate (30)
tert-Butyl (2-(2-nitro-1*H*-imidazol-1-yl)ethyl)carbamate (31)
2-(2-Nitro-1*H*-imidazol-1-yl)ethan-1-amine (32)
N-(2-Methoxyethyl)(2-nitro-1*H*-imidazol-1-yl)methanesulfonamide (33)
N-(2-Morpholinoethyl)-1-(2-nitro-1*H*-imidazol-1-yl)methanesulfonamide (34)
N-(2-Methoxyethyl)-2-(2-nitro-1*H*-imidazol-1-yl)ethanesulfonamide (35)
N-[2-(4-Morpholinyl)ethyl]-2-(2-nitro-1*H*-imidazol-1-yl)ethanesulfonamide (36)
N-(2-Methoxyethyl)(5-nitro-1*H*-imidazol-1-yl)methanesulfonamide (37)
N-(2-Morpholinoethyl)-1-(5-nitro-1*H*-imidazol-1-yl)methanesulfonamide (38)
N-(2-Methoxyethyl)-2-(5-nitro-1*H*-imidazol-1-yl)ethane-1-sulfonamide (39)
N-(2-Methoxyethyl)-2-(2-methyl-5-nitro-1*H*-imidazol-1-yl)ethanesulfonamide (40)

N-(2-Hydroxyethyl)-2-(2-methyl-5-nitro-1*H*-imidazol-1-yl)ethanesulfonamide (41)
2-(2-Methyl-5-nitro-1*H*-imidazol-1-yl)-*N*-[2-(4-morpholinyl)ethyl]ethanesulfonamide (42)
1-Methyl-4-{[2-(2-methyl-5-nitro-1*H*-imidazol-1-yl)ethyl]sulfonyl}piperazine (43)
2-(2-Methyl-5-nitro-1*H*-imidazol-1-yl)-*N*-[3-(4-morpholinyl)propyl]ethanesulfonamide (44)
2-(2-Methyl-5-nitro-1*H*-imidazol-1-yl)-*N*-[2-(1-piperidinyl)ethyl]ethanesulfonamide (45)
N,*N*-Dimethyl-1-{[2-(2-methyl-5-nitro-1*H*-imidazol-1-yl)ethyl]sulfonyl}-4-piperidinamine (46)

Compound 13 1H

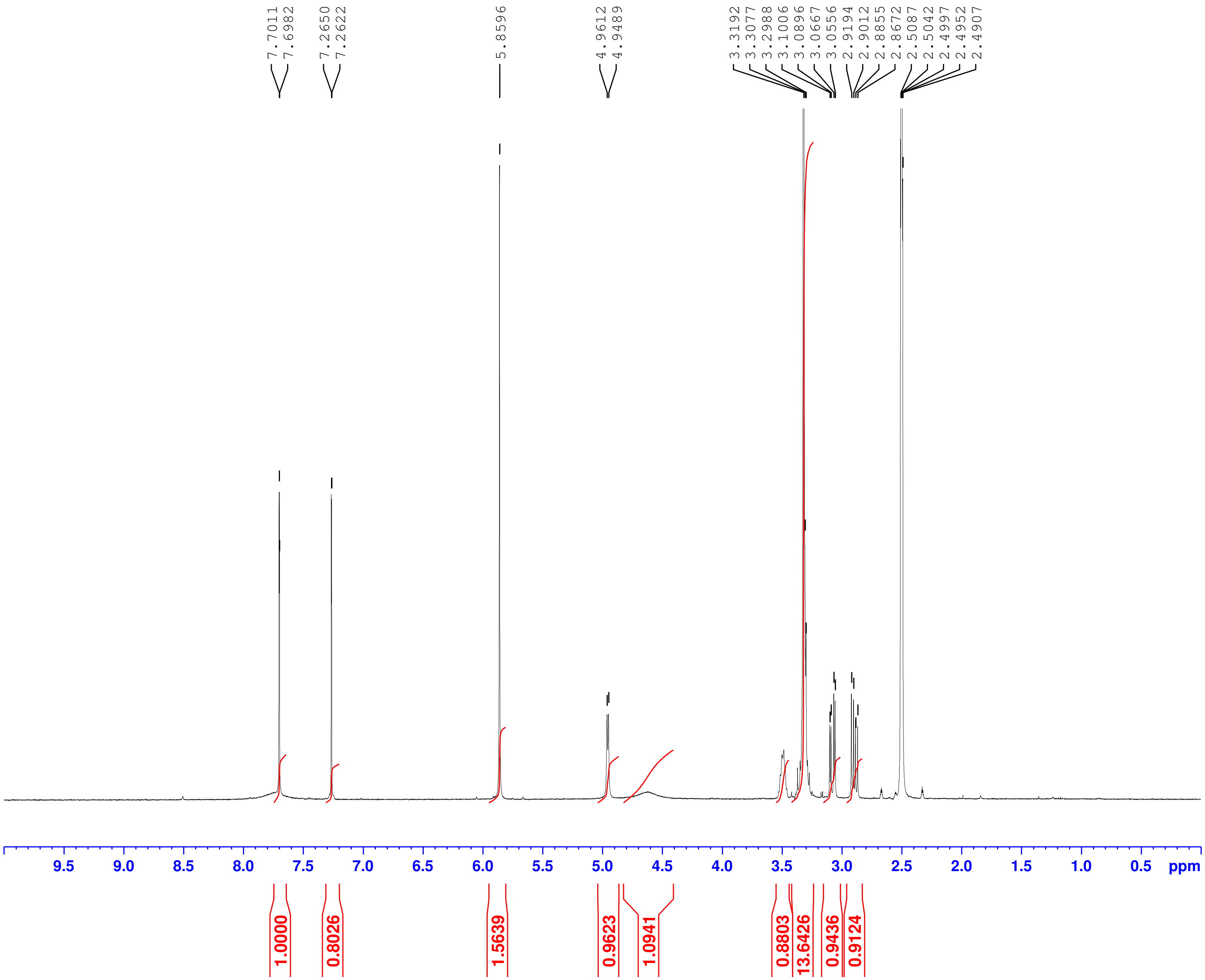

Current Data Parameters
NAME Dec12-2017
EXPNO 3
PROCNO 1

F2 - Acquisition Parameters
Date_ 20171212
Time 9.40 h
INSTRUM spect
PROBHD Z108618_0860 (
PULPROG zg30
TD 65536
SOLVENT DMSO
NS 64
DS 2
SWH 8012.820 Hz
FIDRES 0.244532 Hz
AQ 4.0894465 sec
RG 198.55
DW 62.400 usec
DE 6.50 usec
TE 298.0 K
D1 1.00000000 sec
TD0 1
SFO1 400.1324708 MHz
NUC1 1H
P1 13.60 usec
PLW1 13.19999981 W

F2 - Processing parameters
SI 65536
SF 400.1300027 MHz
WDW EM
SSB 0
LB 0.30 Hz
GB 0
PC 1.00

Compound 13 13C

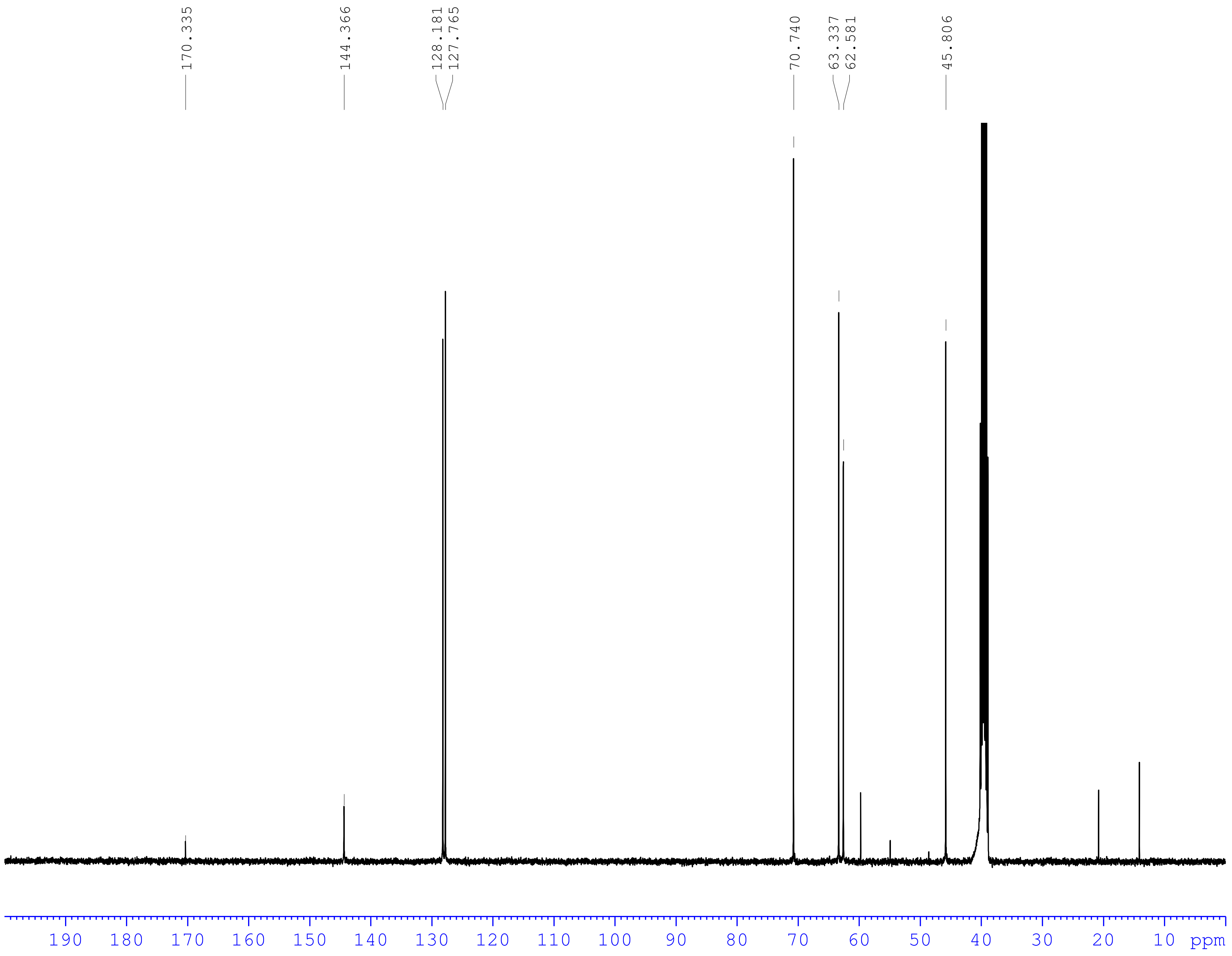

Current Data Parameters
NAME Jul29-2014-FMHSacsrcnmr
EXPNO 25
PROCNO 1

F2 - Acquisition Parameters
Date_ 20140730
Time 6.16 h
INSTRUM spect
PROBHD 5 mm BBO BB-1H
PULPROG zgpg50
TD 65536
SOLVENT DMSO
NS 24000
DS 4
SWH 26178.008 Hz
FIDRES 0.798889 Hz
AQ 1.2517377 sec
RG 11585.2
DW 19.100 usec
DE 10.00 usec
TE 298.0 K
D1 0.75000000 sec
d11 0.03000000 sec
DELTA 0.64999998 sec
TD0 1
SFO1 100.6248351 MHz
NUC1 13C
P1 11.80 usec
SFO2 400.1316677 MHz
NUC2 1H
CPDPRG[2 waltz16
PCPD2 100.00 usec

F2 - Processing parameters
SI 32768
SF 100.6128165 MHz
WDW EM
SSB 0
LB 1.00 Hz
GB 0
PC 1.40

Compound 13

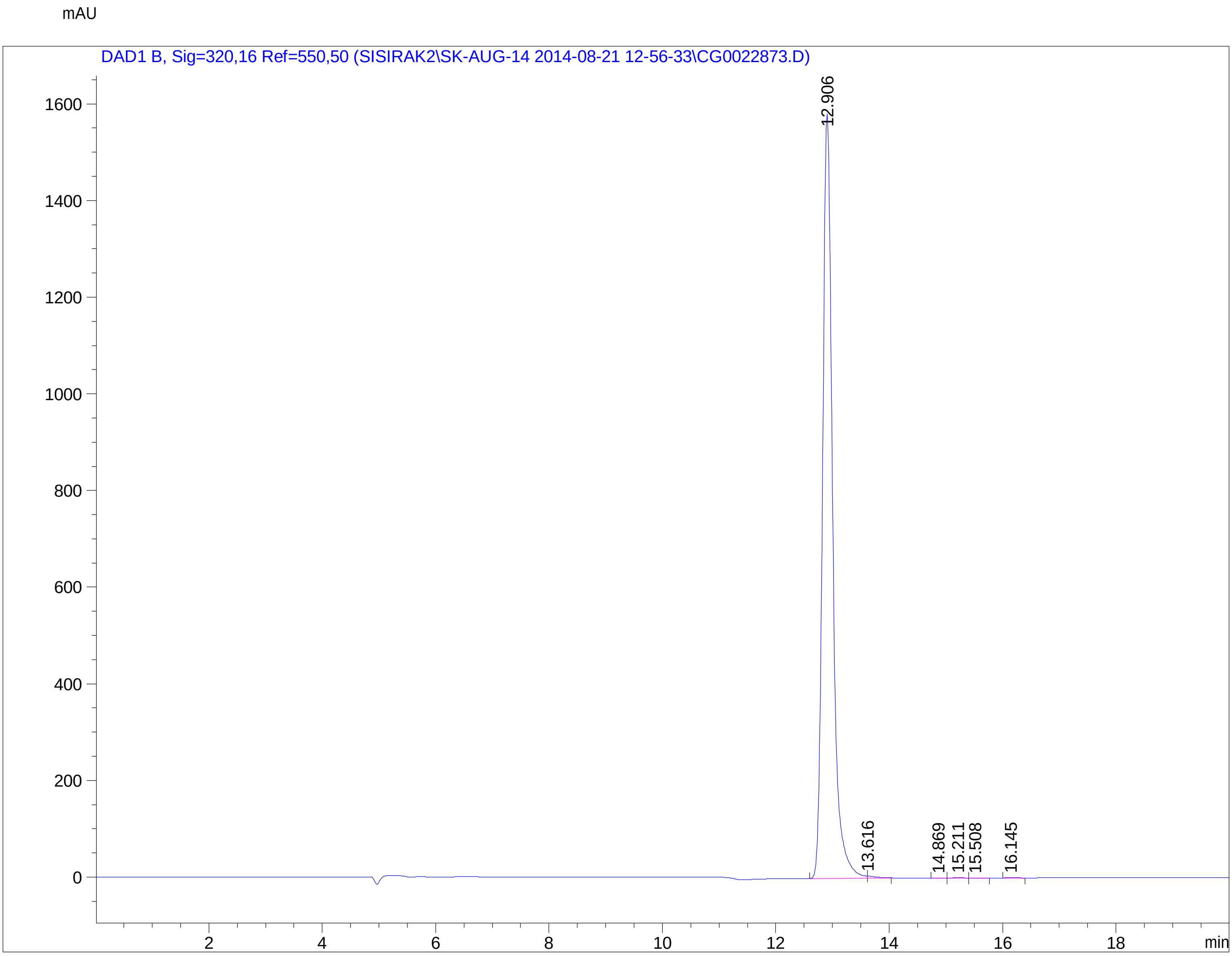

Area Percent Report

Signal 1: DAD1 B, Sig=320,16 Ref=550,50

Peak #	RetTime [min]	Type	Width [min]	Area [mAU*s]	Height [mAU]	Area %
1	12.906	MF	0.2036	1.93487e4	1584.10071	99.5742
2	13.616	FM	0.1802	47.05440	4.35176	0.2422
3	14.869	MF	0.1476	3.03744	3.42872e-1	0.0156
4	15.211	MF	0.1794	16.90825	1.57065	0.0870
5	15.508	FM	0.2148	4.59533	3.56635e-1	0.0236
6	16.145	MM	0.1998	11.15317	9.30451e-1	0.0574

Compound 14 1H

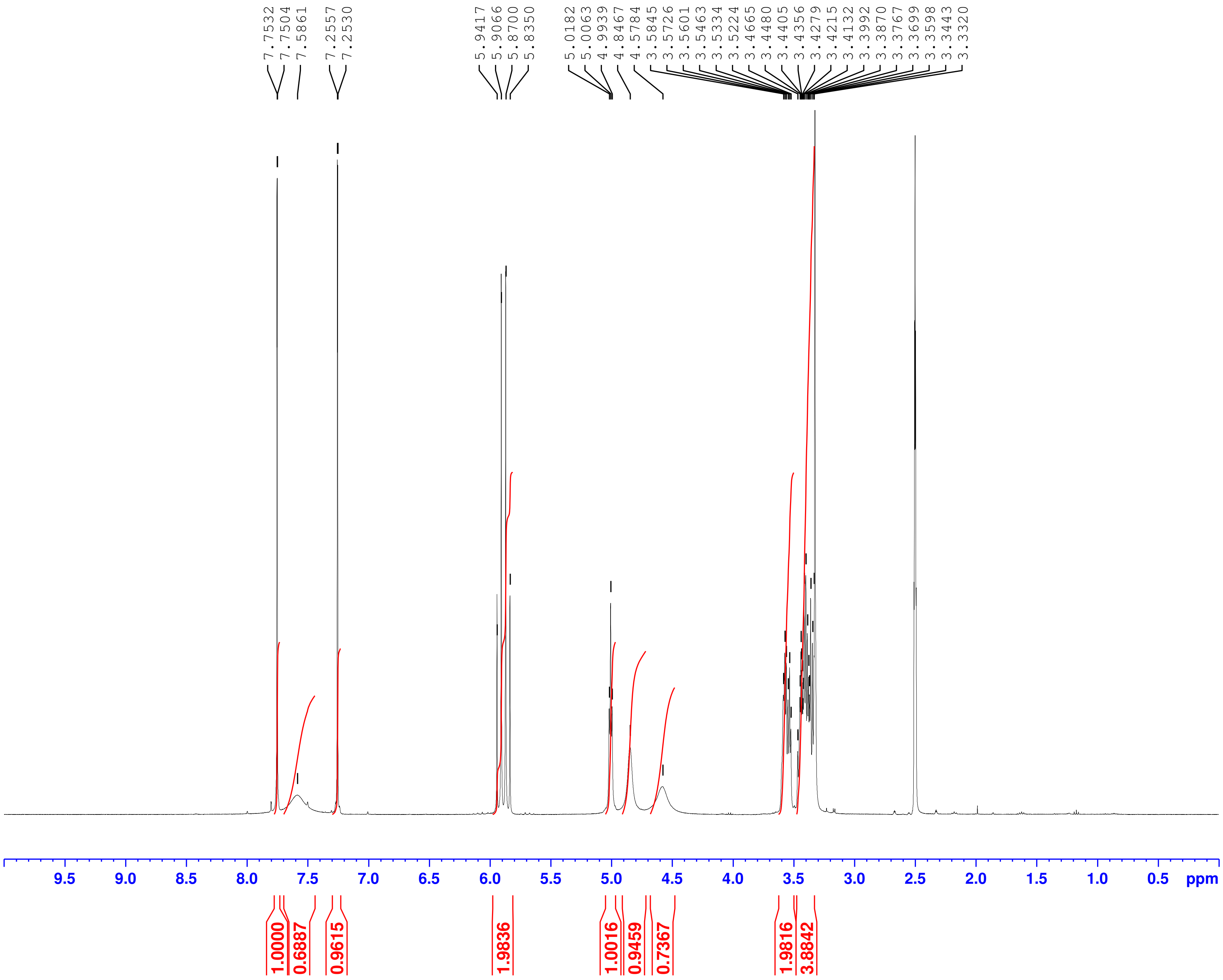

Current Data Parameters
NAME Jan24-2018
EXPNO 16
PROCNO 1

F2 - Acquisition Parameters
Date_ 20180124
Time 16.47 h
INSTRUM spect
PROBHD Z108618_0860 (
PULPROG zg30
TD 65536
SOLVENT DMSO
NS 64
DS 2
SWH 8012.820 Hz
FIDRES 0.244532 Hz
AQ 4.0894465 sec
RG 156.54
DW 62.400 usec
DE 6.50 usec
TE 298.0 K
D1 1.00000000 sec
TD0 1
SFO1 400.1324708 MHz
NUC1 1H
P1 13.60 usec
PLW1 13.19999981 W

F2 - Processing parameters
SI 65536
SF 400.1300027 MHz
WDW EM
SSB 0
LB 0.30 Hz
GB 0
PC 1.00

Compound 14 13C

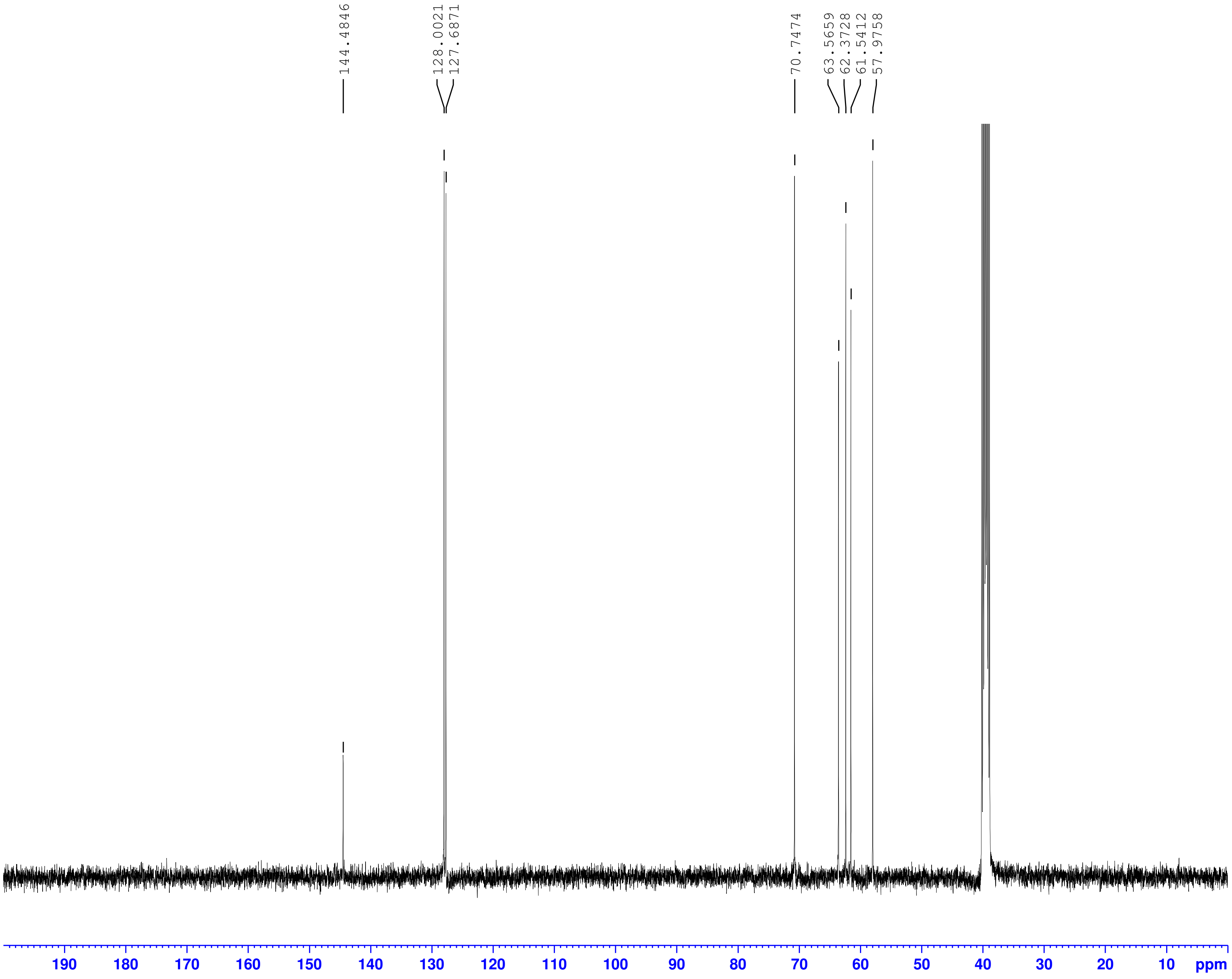


```
Current Data Parameters
NAME          Jan24-2018
EXPNO                 20
PROCNO                 1

F2 - Acquisition Parameters
Date_           20180125
Time                0.52 h
INSTRUM            spect
PROBHD    Z108618_0860 (
PULPROG           zgpg50
TD                 65536
SOLVENT             DMSO
NS                  6000
DS                     4
SWH            24038.461 Hz
FIDRES          0.733596 Hz
AQ             1.3631488 sec
RG                198.55
DW                20.800 usec
DE                  6.50 usec
TE                 298.0 K
D1            0.63999999 sec
D11           0.03000000 sec
TD0                    1
SFO1         100.6228298 MHz
NUC1                 13C
P1                 10.00 usec
PLW1         48.17399979 W
SFO2         400.1316005 MHz
NUC2                  1H
CPDPRG[2         waltz16
PCPD2              90.00 usec
PLW2         13.19999981 W
PLW12         0.30142000 W
PLW13         0.15161000 W

F2 - Processing parameters
SI                 32768
SF           100.6128156 MHz
WDW                   EM
SSB                    0
LB                  1.00 Hz
GB                     0
PC                  1.40
```

Compound 14

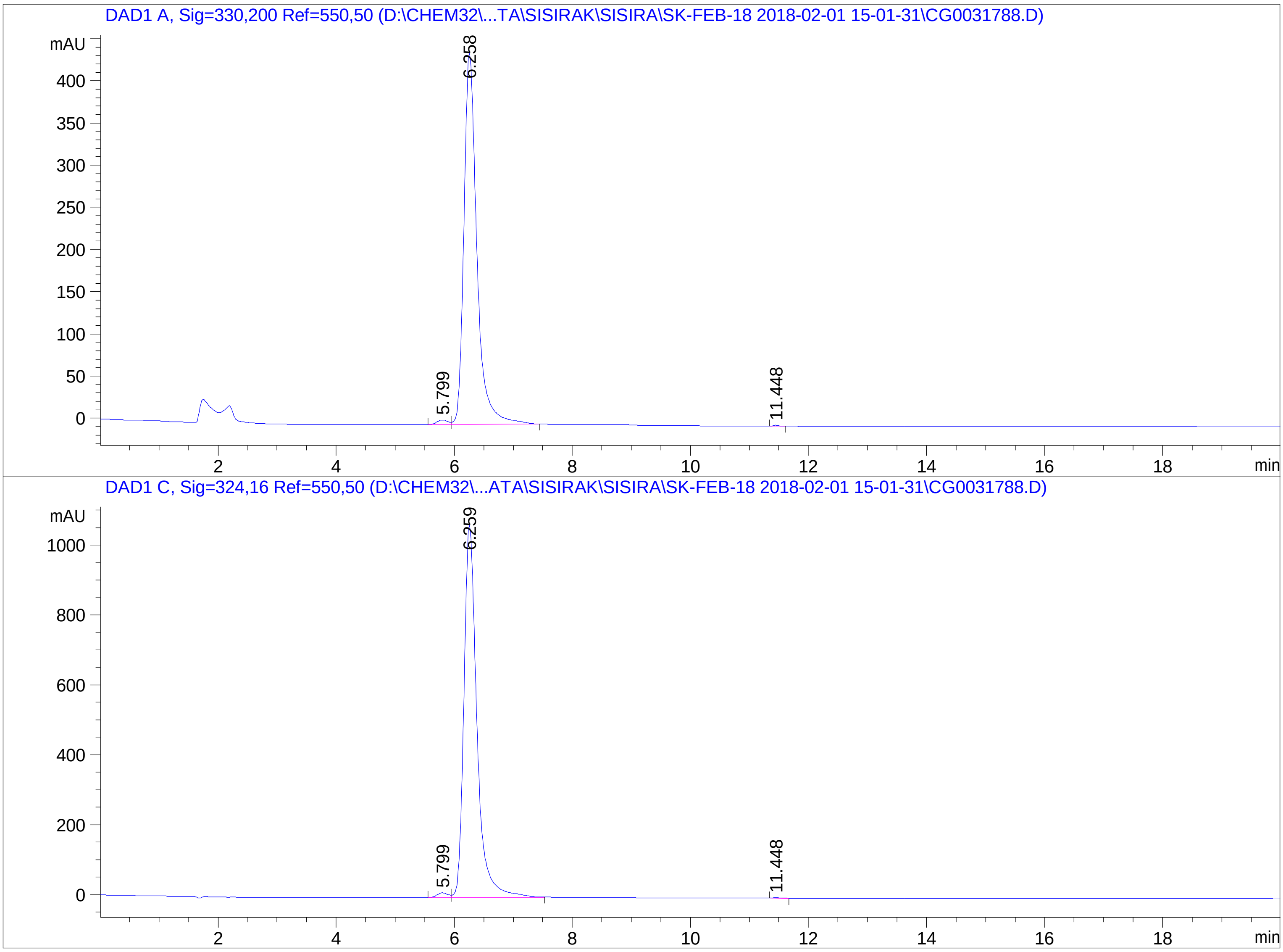

Signal 1: DAD1 A, Sig=330,200 Ref=550,50

Peak #	RetTime [min]	Type	Width [min]	Area [mAU*s]	Height [mAU]	Area %
1	5.799	BV	0.1955	68.46997	5.41624	1.0085
2	6.258	VB	0.2300	6712.61426	440.04041	98.8701
3	11.448	BB	0.1051	8.24262	1.18538	0.1214

Totals : 6789.32685 446.64203

Compound 15 1H

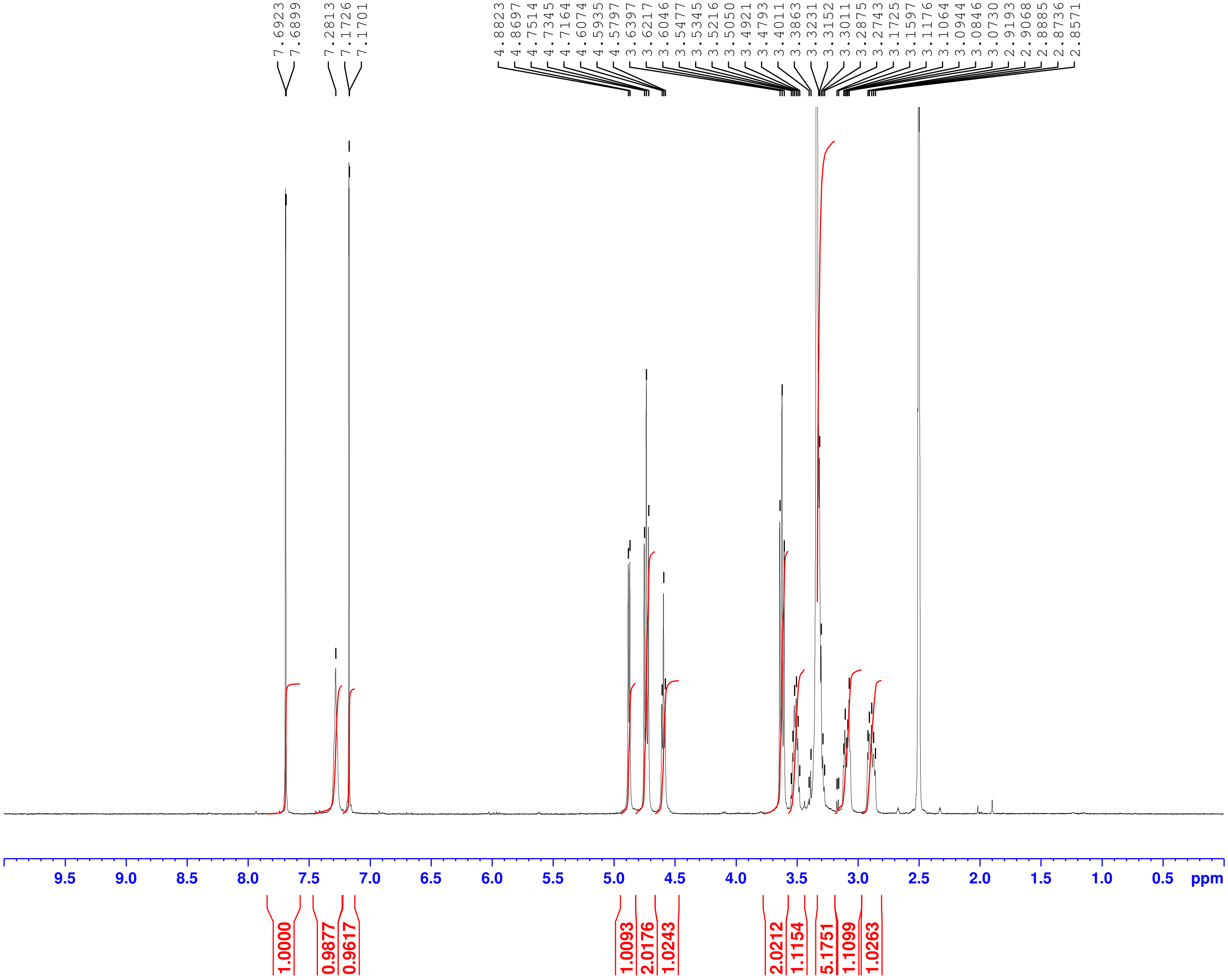


```
Current Data Parameters
NAME          May05-2023
EXPNO                  5
PROCNO                 1

F2 - Acquisition Parameters
Date_           20230505
Time               15.08 h
INSTRUM            spect
PROBHD   Z108618_0860 (
PULPROG             zg30
TD                 65536
SOLVENT             DMSO
NS                    12
DS                     2
SWH             8012.820 Hz
FIDRES          0.244532 Hz
AQ             4.0894465 sec
RG                156.54
DW                62.400 usec
DE                  6.50 usec
TE                 298.0 K
D1            1.00000000 sec
TD0                    1
SFO1         400.1324708 MHz
NUC1                  1H
P0                  4.53 usec
P1                 13.60 usec
PLW1         13.19999981 W

F2 - Processing parameters
SI                 65536
SF           400.1300025 MHz
WDW                   EM
SSB                    0
LB                  0.30 Hz
GB                     0
PC                  1.00
```

Compound 15 13C

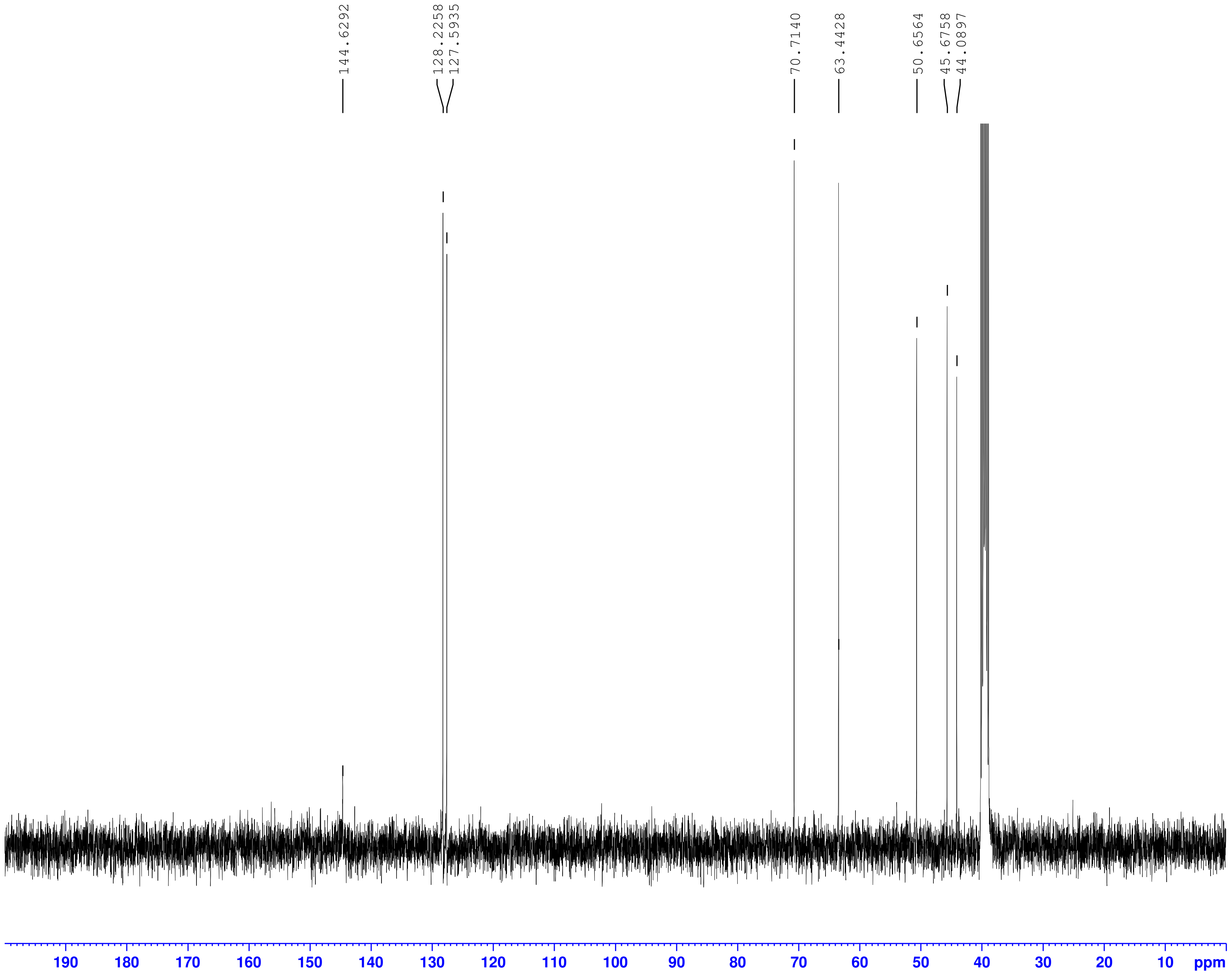

Current Data Parameters
NAME May05-2023
EXPNO 6
PROCNO 1

F2 - Acquisition Parameters
Date_ 20230505
Time 15.41 h
INSTRUM spect
PROBHD Z108618_0860 (
PULPROG zgpg50
TD 65536
SOLVENT DMSO
NS 917
DS 4
SWH 24038.461 Hz
FIDRES 0.733596 Hz
AQ 1.3631488 sec
RG 198.55
DW 20.800 usec
DE 6.50 usec
TE 298.0 K
D1 0.63999999 sec
D11 0.03000000 sec
TD0 1
SFO1 100.6228298 MHz
NUC1 13C
P1 10.00 usec
PLW1 48.17399979 W
SFO2 400.1316005 MHz
NUC2 1H
CPDPRG[2 waltz16
PCPD2 90.00 usec
PLW2 13.19999981 W
PLW12 0.30142000 W
PLW13 0.15161000 W

F2 - Processing parameters
SI 32768
SF 100.6128156 MHz
WDW EM
SSB 0
LB 1.00 Hz
GB 0
PC 1.40

Compound 15

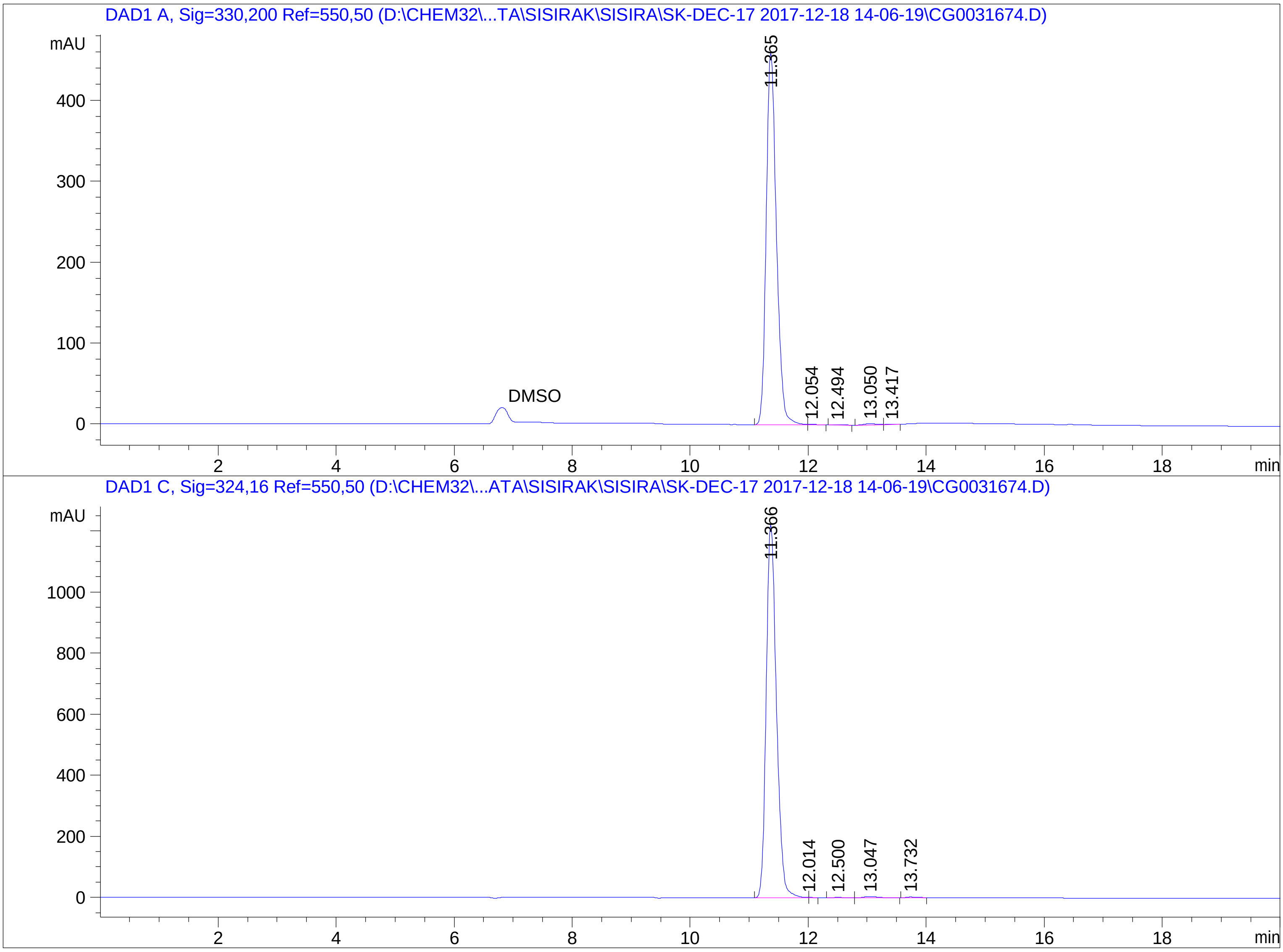

Signal 1: DAD1 A, Sig=330,200 Ref=550,50

Peak #	RetTime [min]	Type	Width [min]	Area [mAU*s]	Height [mAU]	Area %
1	11.365	MF	0.1991	5511.03223	461.43466	99.2574
2	12.054	FM	0.1702	7.18620	7.03511e-1	0.1294
3	12.494	MM	0.1868	5.84591	5.21696e-1	0.1053
4	13.050	MF	0.2527	24.20798	1.59655	0.4360
5	13.417	FM	0.2131	3.98964	3.12028e-1	0.0719

Totals : 5552.26196 464.56844

Compound 16 1H

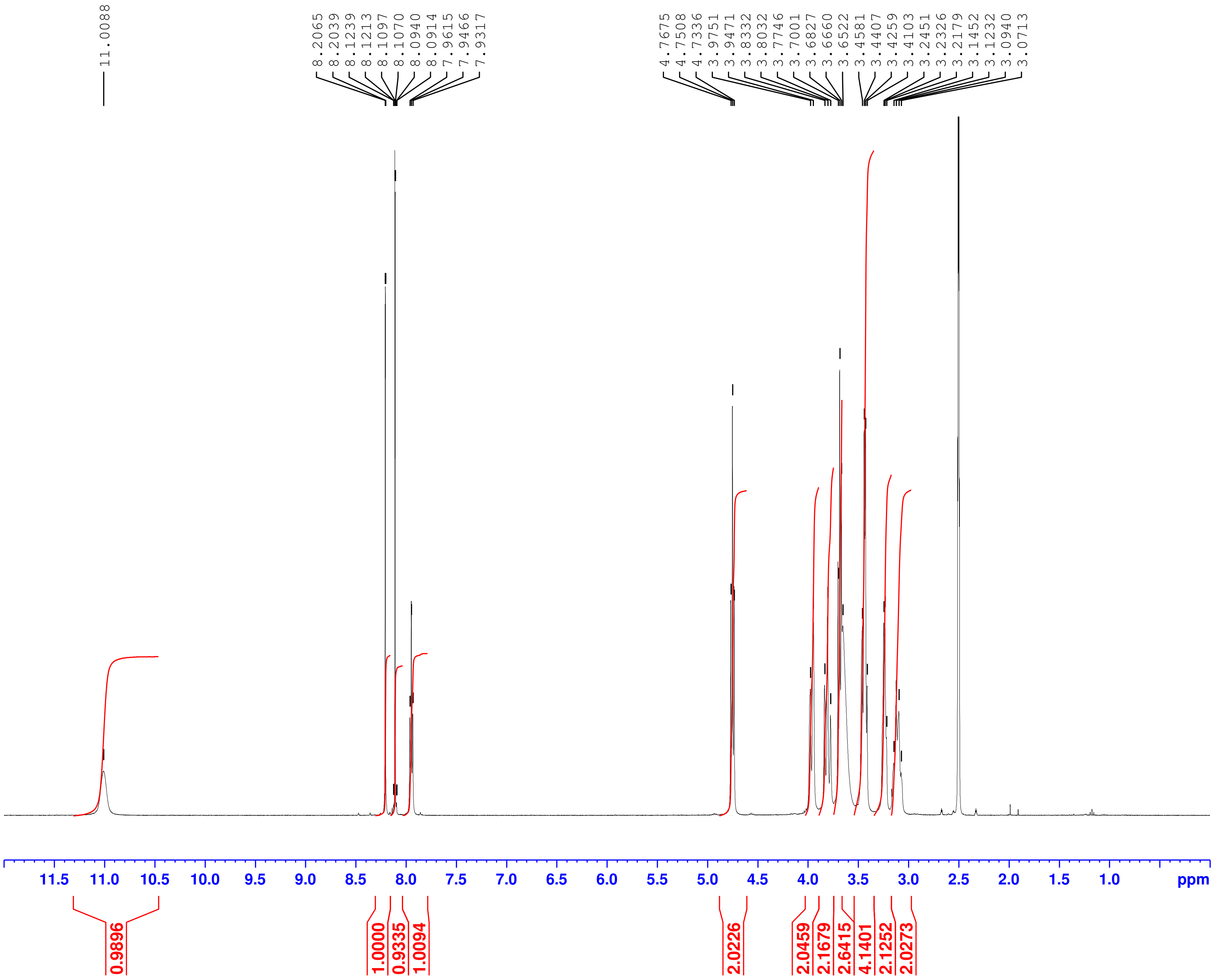

Current Data Parameters
NAME Nov17-2017
EXPNO 21
PROCNO 1

F2 - Acquisition Parameters
Date_ 20171118
Time 16.46 h
INSTRUM spect
PROBHD Z108618_0860 (
PULPROG zg30
TD 65536
SOLVENT DMSO
NS 64
DS 2
SWH 8012.820 Hz
FIDRES 0.244532 Hz
AQ 4.0894465 sec
RG 176.55
DW 62.400 usec
DE 6.50 usec
TE 298.0 K
D1 1.00000000 sec
TD0 1
SFO1 400.1324708 MHz
NUC1 1H
P1 13.60 usec
PLW1 13.19999981 W

F2 - Processing parameters
SI 65536
SF 400.1300026 MHz
WDW EM
SSB 0
LB 0.30 Hz
GB 0
PC 1.00

Compound 16 13C

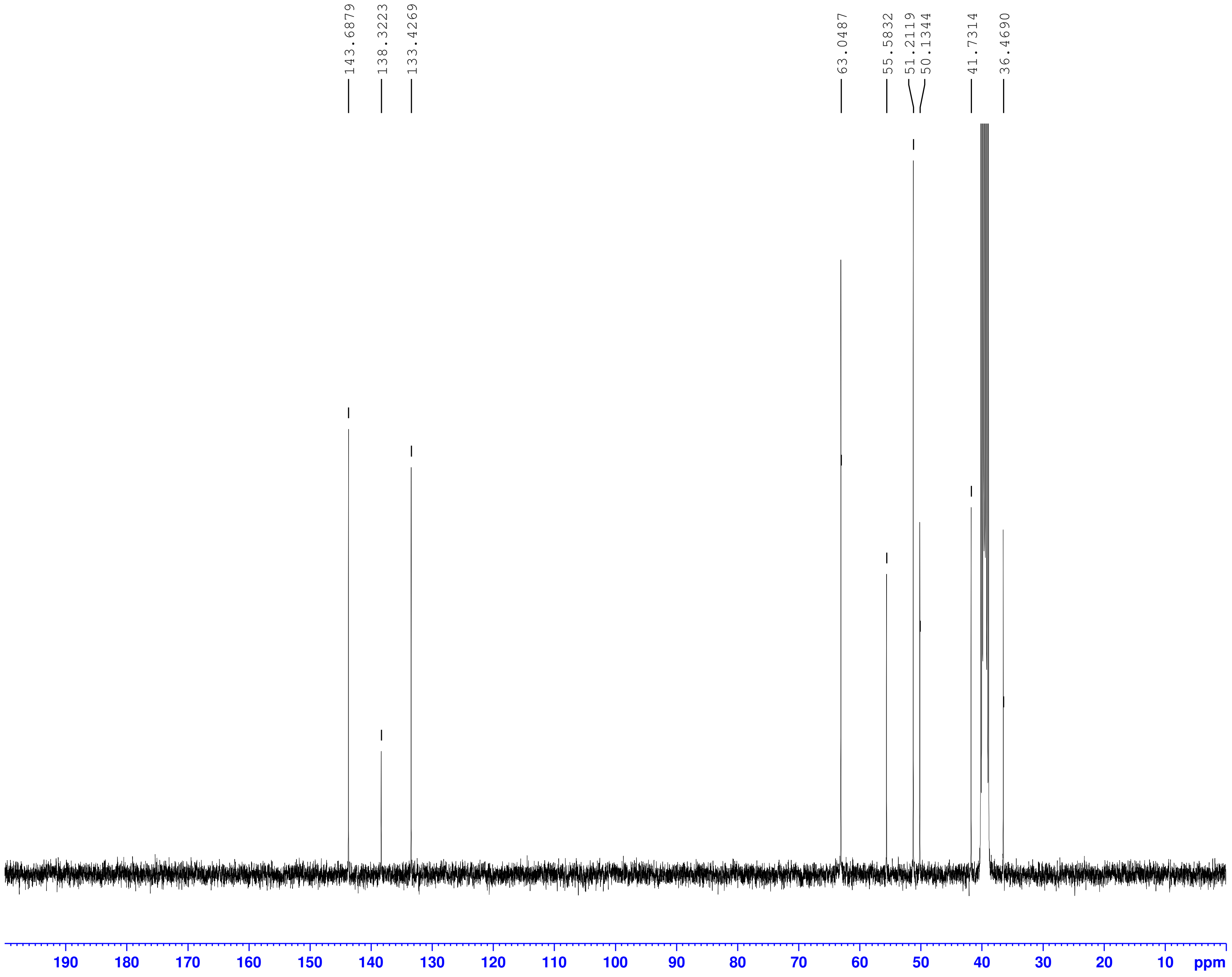

Current Data Parameters
NAME Nov17-2017
EXPNO 22
PROCNO 1

F2 - Acquisition Parameters
Date_ 20171118
Time 20.14 h
INSTRUM spect
PROBHD Z108618_0860 (
PULPROG zgpg50
TD 65536
SOLVENT DMSO
NS 6000
DS 4
SWH 24038.461 Hz
FIDRES 0.733596 Hz
AQ 1.3631488 sec
RG 198.55
DW 20.800 usec
DE 6.50 usec
TE 298.0 K
D1 0.63999999 sec
D11 0.03000000 sec
TD0 1
SFO1 100.6228298 MHz
NUC1 13C
P1 10.00 usec
PLW1 48.17399979 W
SFO2 400.1316005 MHz
NUC2 1H
CPDPRG[2 waltz16
PCPD2 90.00 usec
PLW2 13.19999981 W
PLW12 0.30142000 W
PLW13 0.15161000 W

F2 - Processing parameters
SI 32768
SF 100.6128175 MHz
WDW EM
SSB 0
LB 1.00 Hz
GB 0
PC 1.40

Compound 16

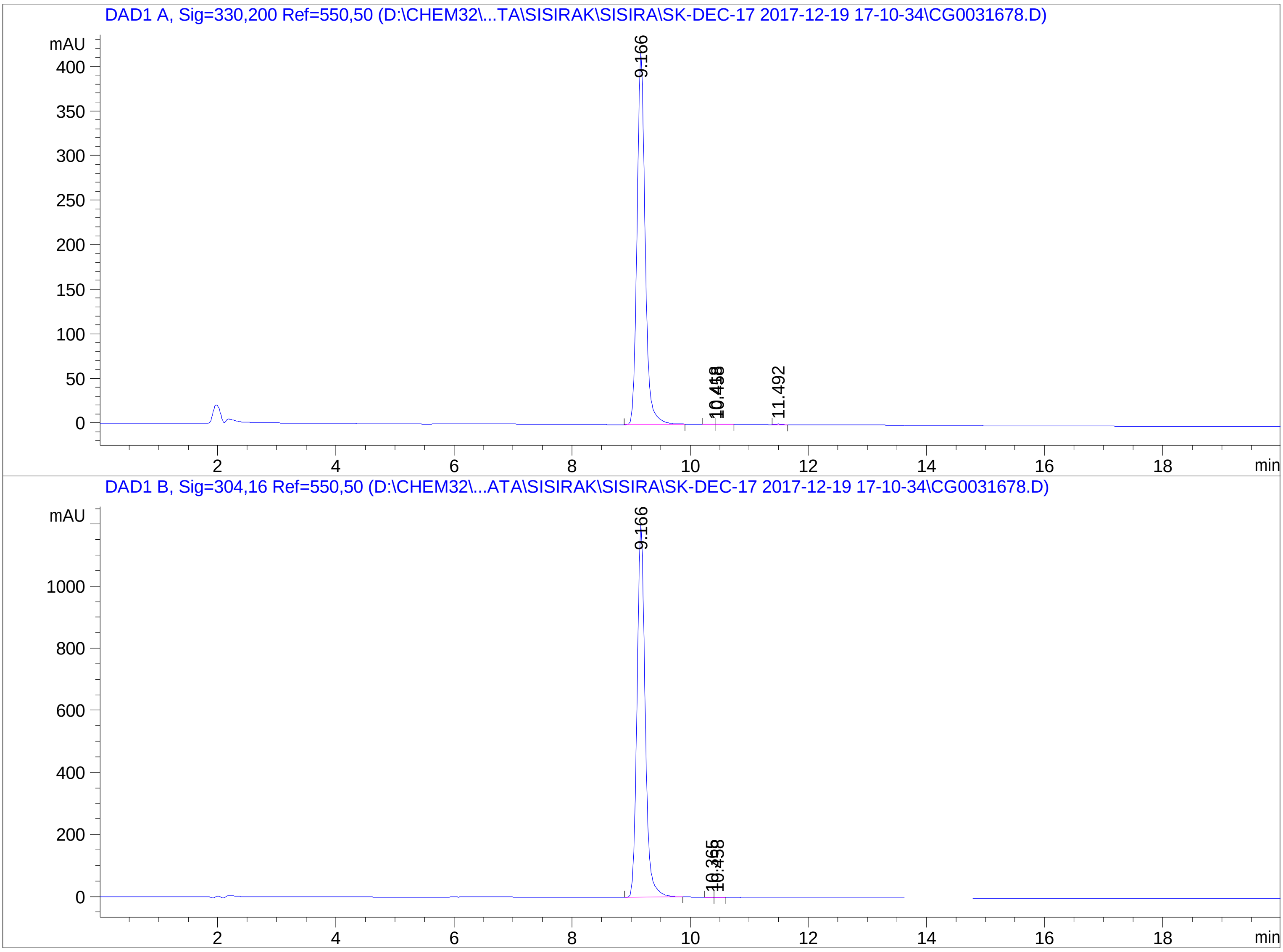

Signal 1: DAD1 A, Sig=330,200 Ref=550,50

Signal 1: DAD1 A, Sig=330,200 Ref=550,50

Peak #	RetTime [min]	Type	Width [min]	Area [mAU*s]	Height [mAU]	Area %
1	9.166	MM	0.1589	3980.00635	417.40576	99.8336
2	10.418	MF	0.1356	1.04060	1.27894e-1	0.0261
3	10.458	FM	0.1459	1.22499	1.39954e-1	0.0307
4	11.492	MM	0.1126	4.36763	6.46343e-1	0.1096

Totals : 3986.63958 418.31995

Compound 17 1H

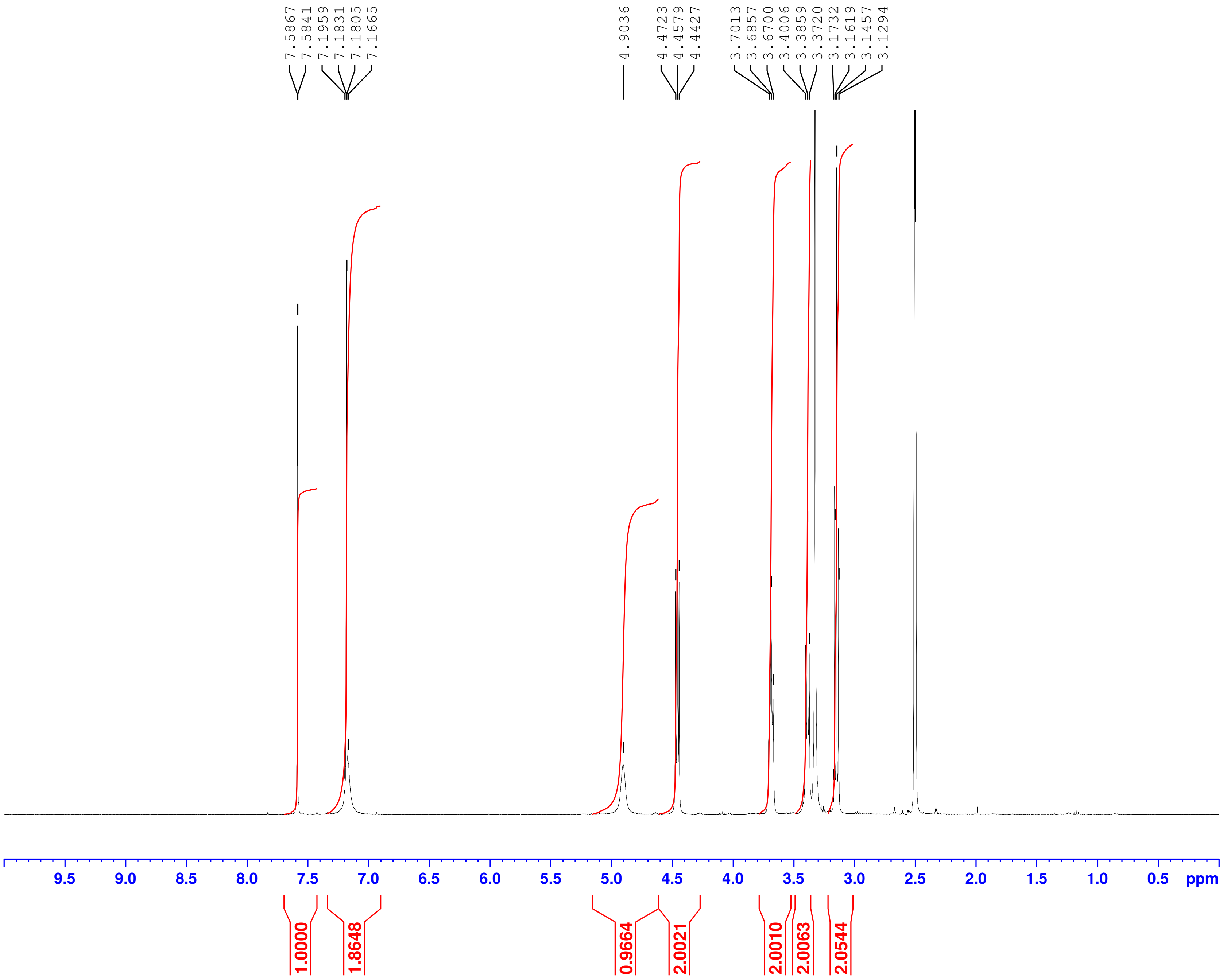


```
Current Data Parameters
NAME          Feb28-2018
EXPNO                  7
PROCNO                 1

F2 - Acquisition Parameters
Date_           20180228
Time               11.39 h
INSTRUM            spect
PROBHD   Z108618_0860 (
PULPROG             zg30
TD                 65536
SOLVENT             DMSO
NS                    64
DS                     2
SWH             8012.820 Hz
FIDRES          0.244532 Hz
AQ             4.0894465 sec
RG                176.55
DW                62.400 usec
DE                  6.50 usec
TE                 298.0 K
D1            1.00000000 sec
TD0                    1
SFO1         400.1324708 MHz
NUC1                  1H
P1                 13.60 usec
PLW1         13.19999981 W

F2 - Processing parameters
SI                 65536
SF           400.1300026 MHz
WDW                   EM
SSB                    0
LB                  0.30 Hz
GB                     0
PC                  1.00
```

Compound 17 13C

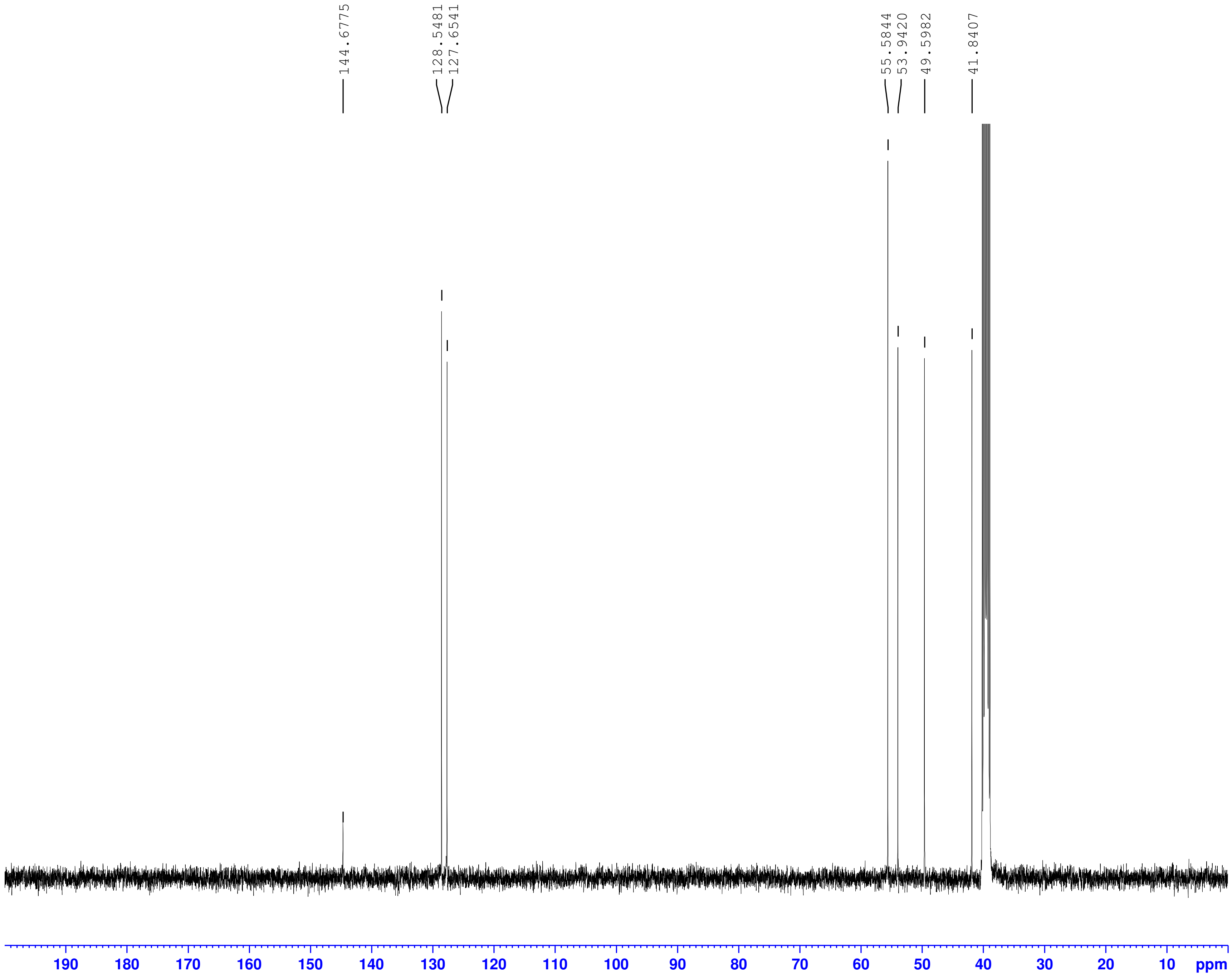


```
Current Data Parameters
NAME          Feb01-2018
EXPNO                 20
PROCNO                 1

F2 - Acquisition Parameters
Date_           20180201
Time               18.47 h
INSTRUM            spect
PROBHD   Z108618_0860 (
PULPROG           zgpg50
TD                 65536
SOLVENT             DMSO
NS                  4000
DS                     4
SWH            24038.461 Hz
FIDRES          0.733596 Hz
AQ             1.3631488 sec
RG                198.55
DW                20.800 usec
DE                  6.50 usec
TE                 298.0 K
D1            0.63999999 sec
D11           0.03000000 sec
TD0                    1
SFO1         100.6228298 MHz
NUC1                 13C
P1                 10.00 usec
PLW1         48.17399979 W
SFO2         400.1316005 MHz
NUC2                  1H
CPDPRG[2         waltz16
PCPD2              90.00 usec
PLW2         13.19999981 W
PLW12         0.30142000 W
PLW13         0.15161000 W

F2 - Processing parameters
SI                 32768
SF           100.6128148 MHz
WDW                   EM
SSB                    0
LB                  1.00 Hz
GB                     0
PC                  1.40
```

Compound 17

Additional Info : Peak(s) manually integrated

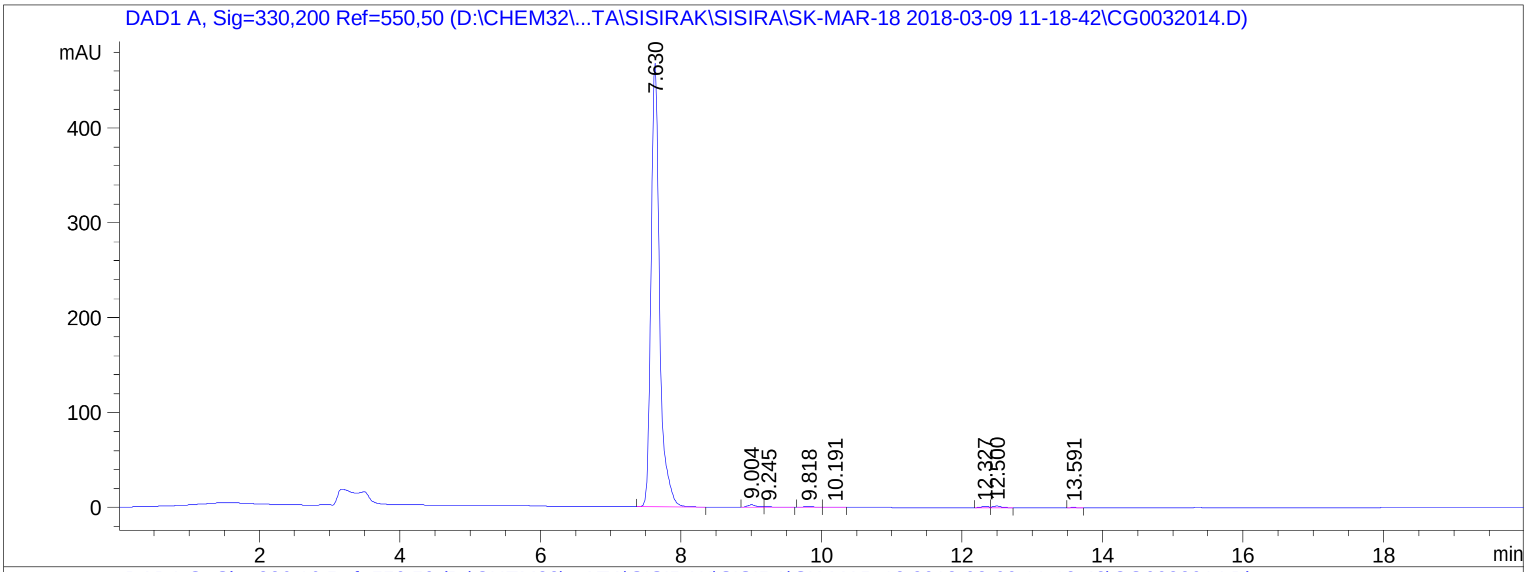

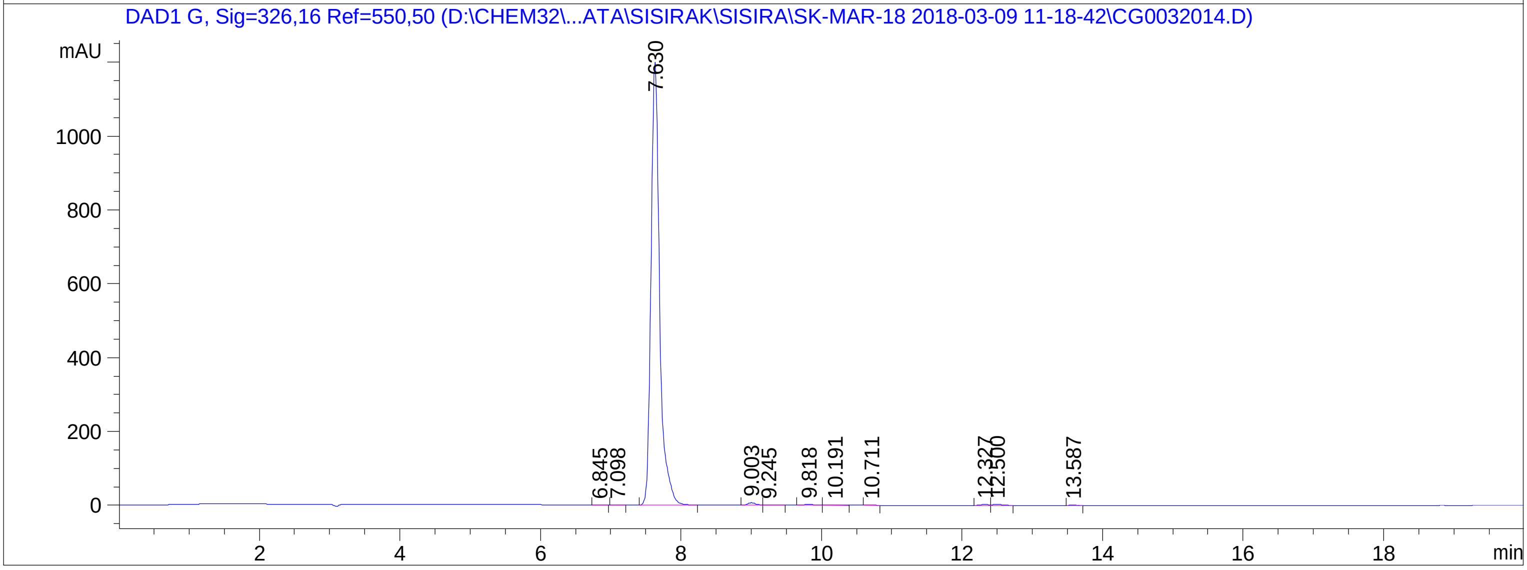

Area Percent Report

Signal 1: DAD1 A, Sig=330,200 Ref=550,50

Peak #	RetTime [min]	Type	Width [min]	Area [mAU*s]	Height [mAU]	Area %
1	7.630	MM	0.1395	3925.43115	469.09988	98.3915
2	9.004	MF	0.1371	21.19360	2.57715	0.5312
3	9.245	FM	0.1430	4.14336	4.82906e-1	0.1039
4	9.818	MF	0.1608	10.54772	1.09347	0.2644
5	10.191	FM	0.1852	4.00984	3.60831e-1	0.1005
6	12.327	BV	0.1165	8.95267	1.18076	0.2244
7	12.500	VB	0.1266	12.95241	1.53330	0.3247
8	13.591	MM	0.1254	2.37355	3.15503e-1	0.0595

Totals : 3989.60430 476.64380
